# Recently Evolved *Francisella*-Like Endosymbiont Outcompetes an Ancient and Evolutionarily Associated *Coxiella*-Like Endosymbiont in the Lone Star Tick (*Amblyomma americanum*) Linked to the Alpha-Gal Syndrome

**DOI:** 10.3389/fcimb.2022.787209

**Published:** 2022-04-12

**Authors:** Deepak Kumar, Surendra Raj Sharma, Abdulsalam Adegoke, Ashley Kennedy, Holly C. Tuten, Andrew Y. Li, Shahid Karim

**Affiliations:** ^1^ School of Biological, Environmental, and Earth Sciences, University of Southern Mississippi, Hattiesburg, MS, United States; ^2^ Delaware Division of Fish & Wildlife, Delaware Mosquito Control Sect., Newark, DE, United States; ^3^ Illinois Natural History Survey (INHS), University of Illinois Urbana-Champaign, Champaign, IL, United States; ^4^ Invasive Insect Biocontrol & Behavior Laboratory, United States Department of Agriculture, Agricultural Research Service (USDA ARS), Beltsville, MD, United States; ^5^ Center for Molecular and Cellular Biosciences, University of Southern Mississippi, Hattiesburg, MS, United States

**Keywords:** alpha-gal syndrome, *Amblyomma americanum*, endosymbionts, lone star tick, microbiome, ticks

## Abstract

**Background:**

Ticks are hematophagous arthropods that transmit various bacterial, viral, and protozoan pathogens of public health significance. The lone star tick (*Amblyomma americanum*) is an aggressive human-biting tick that transmits bacterial and viral pathogens, and its bites are suspected of eliciting the alpha-gal syndrome, a newly emerged delayed hypersensitivity following consumption of red meat in the United States. While ongoing studies have attempted to investigate the contribution of different tick-inherent factors to the induction of alpha-gal syndrome, an otherwise understudied aspect is the contribution of the tick microbiome and specifically obligate endosymbionts to the establishment of the alpha-gal syndrome in humans.

**Materials and Methods:**

Here we utilized a high-throughput metagenomic sequencing approach to cataloging the entire microbial communities residing within different developmental stages and tissues of unfed and blood-fed ticks from laboratory-maintained ticks and three new geographical locations in the United States. The Quantitative Insights Into Microbial Ecology (QIIME2) pipeline was used to perform data analysis and taxonomic classification. Moreover, using a SparCC (Sparse Correlations for Compositional data) network construction model, we investigated potential interactions between members of the microbial communities from laboratory-maintained and field-collected ticks.

**Results:**

Overall, Francisellaceae was the most dominant bacteria identified in the microbiome of both laboratory-raised and field-collected *Am. americanum* across all tissues and developmental stages. Likewise, microbial diversity was seen to be significantly higher in field-collected ticks compared with laboratory-maintained ticks as seen with a higher number of both Operational Taxonomic Units and measures of species richness. Several potential positive and negative correlations were identified from our network analysis. We observed a strong positive correlation between Francisellaceae, Rickettsiaceae, and Midichloriaceae in both developmental stages and tissues from laboratory-maintained ticks, whereas ovarian tissues had a strong positive correlation of bacteria in the family Xanthobacteraceae and Rhizobiaceae. A negative interaction was observed between Coxiellaceae and Francisellaceae in Illinois, and all the bacteria detected from ticks from Delaware were negatively correlated.

**Conclusion:**

This study is the first to catalog the microbiome of *Am. americanum* throughout its developmental stages and different tissue niches and report the potential replacement of Coxiellaceae by Francisellaceae across developmental stages and tissues tested except in ovarian tissues. These unique and significant findings advance our knowledge and open a new avenue of research to further understand the role of tick microbiome in tick-borne diseases and develop a holistic strategy to control alpha-gal syndrome.

## Introduction

The lone star tick (*Amblyomma americanum*) is an aggressive and generalist hematophagous species known as a vector of a variety of viral (Heartland virus) and bacterial pathogens (*Ehrlichia chaffeensis*, *Ehrlichia ewingii*, *Francisella tularensis*, and *Borrelia lonestari*) and alpha-gal syndrome (AGS) (Crispell et al., 2019; Sharma et al., 2021). Microorganisms that occupy an arthropod tick vector are collectively called the tick microbiome; however, the collection of commensals, symbiotic, and pathogenic microbes associated with ticks is more precisely termed the “pathobiome”. Past investigations of the pathobiome of the lone star tick primarily focused on field-collected tick samples (nymphs or adults) using the 16S ribosomal RNA (16S rRNA) sequencing approach (Menchaca et al., 2013; Williams-Newkirk et al., 2014; Fryxell and DeBruyn, 2016; Brinkerhoff et al., 2020). Microbes coexisting association with known pathogens within the ticks might influence pathogen infection and transmission. For instance, rickettsial endosymbionts are thought to alter the transmission of other rickettsial pathogens, as reported by the inverse relationship between the infection prevalence of *Rickettsia rickettsii* (pathogen) and *Rickettsia peacockii* (symbiont) in *Dermacentor andersoni* (Burgdorfer et al., 1983). Similarly, the presence of *Coxiella*-related symbionts in the salivary glands (SGs) of *Am. americanum* ticks impairs the transmission of *E. chaffeensis* (Klyachko et al., 2007). In addition to symbionts, ticks maintain a natural bacterial flora predominantly that comprised bacteria from the Proteobacteria, Firmicutes, and Bacteroides phyla (Budachetri et al., 2014; Narasimhan et al., 2014; Budachetri et al., 2016; Budachetri et al., 2017; Karim et al., 2017; Adegoke et al., 2020), which have also been implicated in interference with tick pathogen infection. For example, *Ixodes scapularis* ticks hatched and raised in a sterile environment showed an altered microbiota, impaired gut integrity, and a reduced ability for *Borrelia burgdorferi* to colonize (Narasimhan et al., 2014). As demonstrated in other arthropod vectors (Cirimotich et al., 2011), altering the tick microbiome may also result in a modulated immune response that could also interfere with pathogen infection and transmission. As microbiomes are an integral part of tick biology, a detailed understanding of (1) microbiome composition, (2) stability, and (3) tripartite interaction with the tick, pathogen, and host will facilitate the discovery of new interventional strategies to limit tick-borne diseases (TBDs).

Most microbiome studies have been performed in whole ticks except in a few studies where tick tissues have been used (Andreotti et al., 2011; Budachetri et al., 2014; Narasimhan et al., 2014; Qiu et al., 2014; Budachetri et al., 2018). Microbiome profiling using whole ticks has limitations, such as difficulty profiling a specific microbial niche at the tissue level (Schabereiter-Gurtner et al., 2003). Profiling of the microbiome associated with vital tissues is necessary for deriving functional inferences of tick biology, TBDs, and interactions (e.g., tick-pathogen, pathogen-endosymbiont) (Narasimhan and Fikrig, 2015). Furthermore, exoskeleton-associated environmental contaminants may not be differentiated from *bona fide* tick tissue–associated bacteria (Narasimhan et al., 2014). Previous studies have also shed light on the prevalence of certain endosymbionts within tick samples. A previously published study showed that approximately one-third of the tick species examined either lacked *Coxiella*-like endosymbiont (CLE) or harbored lower frequencies than expected of an obligate endosymbiont (Duron et al., 2014; Duron et al., 2015). Tick species deficient in CLE are scattered among major tick families and genera, suggesting its frequent and repeated loss during tick evolution (Duron et al., 2015). The possible role of CLE in nutrition, osmoregulation or excretion, synthesis of amino acids, and major B vitamins has been proposed (Klyachko et al., 2007; Machado-Ferreira et al., 2011; Lalzar et al., 2014). Exclusion symbiosis and acquisition of *Francisella*-like endosymbiont (FLE) and *Rickettsia* spp. in ticks could also be speculated as a more efficient alternative for synthesizing B_7_ and B_9_ vitamins (Hunter et al., 2015; Duron et al., 2017) because of the reduced genome of CLE (Gerhart et al., 2016). Other possible factors could be geography, lateral gene transfer, or tick feeding on a shared vertebrate host. There is a gap in our understanding of the ecological factors that could facilitate horizontal transfer (HT) of endosymbionts among tick species. The examination of internal tick organs including SGs has indicated a high concentration of CLEs (Klyachko et al., 2007; Qiu et al., 2014); therefore, feeding on a shared vertebrate host could be an important determinant for HT.

In this study, the microbiome of the lone star tick was determined by using the 16S rRNA sequencing approach. The aims of this study were (a) to determine the composition of the microbiome across the life cycle in unfed (UF) and blood-fed immature and mature tick developmental stages, (*b*) to assess the stability of the microbiome in UF and blood-fed individually dissected tissues (SGs, midgut [MG], and ovary), (*c*) to assess the microbiome composition of field-collected ticks and, (d) to test and validate the hypothesis of acquisition of FLE at the expense of CLE.

## Materials and Methods

### Ticks and Animals

#### Laboratory-Reared Ticks

Laboratory-reared lone star ticks (*Am. americanum*) used in this study were purchased from the Oklahoma State University Tick Rearing Facility. Adult ticks were infested on sheep and allowed to feed until repletion. Engorged ticks were cleaned with 70% ethanol and placed separately in sterile vials and kept in an incubator according to standard practices for maintaining sterile condition (Patrick and Hair, 1975) at 80% to 90% RH under a 14-/10-h light-dark photoperiod at 27°C ± 2°C until they laid eggs. Then, eggs were transferred to a sterile vial and kept in an incubator under the same conditions until they hatched. The larvae and nymphs were fed on hamster until detachment and kept in the incubator under the same conditions as mentioned previously to allow them to molt into the next developmental stage (nymph and adults, respectively). Newly molted adults were then fed on a rabbit, and partially blood-fed males and females were pulled after 5 days for microbiome analysis. Newly molted larvae, nymphs, and adults were used in this study to ensure that bacteria were not acquired from the environment. The protocol for all animal experiments was approved by the Institutional Animal Care and Use Committee of The University of Southern Mississippi.

#### Field-Collected Ticks

**Figure 1:**
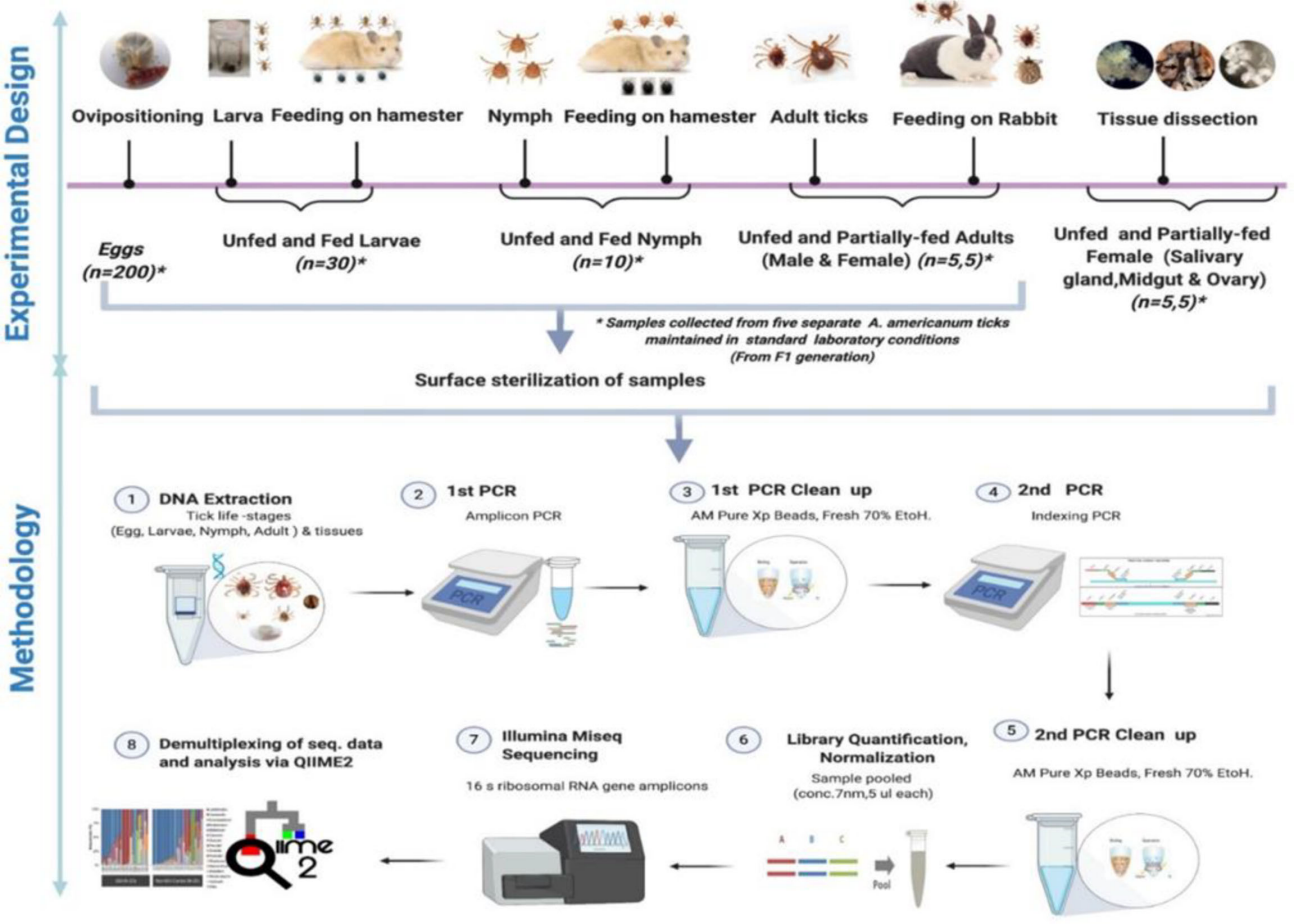
Schematic workflow of microbiome study of the lone star tick for its life stages and tissues.

Questing nymphal and adult stages of the lone star tick were collected by dragging vegetation in Delaware state parks (New Castle, Kent, and Sussex Counties) in June–July 2020 with a 1-m^2^ white flannel drag cloth. Ticks were also collected alive into 14-mL plastic centrifuge tubes in Brown county in June–June *via* dragging of vegetation with 1-m^2^ cloth drags and in DeWitt county with dry-ice baited traps. Ticks were identified alive at the INHS Medical Entomology Lab, and all nymphal and adult (female and male) *Am. americanum* collected were shipped in secured tubes for microbiome analysis. Questing nymphs and adult lone star ticks were similarly collected from vegetations in field locations in Maryland and Illinois. **Figure 1** shows the detailed experimental design.

### Tick Life Stages and DNA Extraction

Five different tick generations were maintained and reared in the laboratory from five separate engorged females. Tick samples from different life stages, namely, egg, larval, nymphal, adult, and various tissues, were collected. All samples (eggs, larvae, nymphs, and adults) were surface sterilized with 2% bleach and washed twice in 70% ethanol for 5 min followed by three washes in sterile deionized water to disinfect the body surface and remove external debris in preparation for DNA extraction. The samples from each developmental stage were divided into five pools of egg clutch (~200 eggs each), 30 UF larvae, 30 fed larvae, 10 UF, and 10 fed nymphs per pool. For microbiome analysis of adult ticks, five separate UF and partially fed (PF) males and female ticks were subjected to DNA extraction. For microbiome analysis of adult female tick tissues, DNA was extracted from dissected tissues (SG, MG, and ovary) of five separate PF and UF female ticks.

The blood-fed female *Am. americanum* were dissected within 4 h of removal from the rabbit. Ticks were dissected, and tissues were collected (SGs, MG, ovary) by following standard procedure (Karim and Ribeiro, 2015). DNA was extracted using a DNeasy Blood and Tissue Kit according to the manufacturer’s protocol (QIAGEN, Germantown, MD, USA). Total DNA was quantified using a Nanodrop spectrophotometer (Thermo Fisher Scientific, USA). The extraction procedures were performed in a biosafety cabinet to ensure that the samples were protected against environmental contamination.

### Library Preparation for 16S Illumina Sequencing and Multiplexing

16S Metagenomic Sequencing Library Preparation protocol from Illumina (San Diego, CA, USA) was followed for library preparation and multiplexing. Dual indexes were used for library preparation. All the primers for amplicon polymerase chain reaction (first PCR) and indexing PCR (second PCR) were ordered from Integrated DNA Technologies (IDT, Coralville, Iowa, USA). Information about all the primers used has been provided in Additional File 1. Multiplexing of tick samples was performed by targeted 16S amplicon sequencing. The gene-specific sequences used in this protocol target the 16S V3 and V4 region. Primers for amplicon PCR were selected from the previously published study (Klindworth et al., 2013). Illumina adapter overhang nucleotide sequences were added to the gene‐specific sequences. The full-length primer sequences for amplicon PCR (first PCR) were 5′-TCGTCGGCAGCGTCAGATGTGTATAAGAGACAGCC TACGGGNGGCWGCAG-3′ (forward), and 5′-GTCTCGTGGGCTCGGAGATGTGTATAAGAGACAGGACTACHVGGGTATCTAATCC-3′ (reverse) where the forward primer overhang sequence was TCGTCGGCAGCGTCAGATGTGTATAAGAGACAG, and the reverse primer overhang sequence was GTCTCGTGGGCTCGGAGATGTGTATAAGAGACAG. The V3–V4 locus-specific forward primer was CCTACGGGNGGCWGCAG, and the reverse primer was GACTACHVGGGTATCTAATCC. As mentioned previously, workflow was based on the 16S Metagenomic Sequencing Library Preparation protocol from Illumina. To determine bias introduced by PCR amplification and sequencing error (Schloss et al., 2011; Schirmer et al., 2015), we included a commercially available Mock Microbial Community Standard, ZymoBIOMICS (catalog #D6306). Dual indexes (i5 and i7) were picked from Nextera Index Kit V2 (Illumina) to individually barcode the amplicons. Specific dual indexes combination for each sample was added to the index PCR (second PCR) primers. After primer designing, amplicon and index PCR primers were ordered from Integrated DNA Technologies (IDT). PCR amplifications and Illumina sequencing were performed for the mock bacterial community in the same manner as for tick samples. Thermocycling conditions for amplicon PCR (first PCR) were 95°C for 3 min, followed by 25 cycles of 95°C for 30 s, 55°C for 30 s, and 72°C for 30 s and a final extension step of 72°C for 5 min. Amplicons were purified with AmPure XP beads (Agencourt Bioscience Corporation, Beverly, MA, USA) as instructed in the manufacturer’s protocol. Purified PCR product was run on 1.5% agarose gel to certify the appropriate size (~500 bps) of amplicon. Purified amplicons were each dissolved in 50 μL of nuclease-free water, and 5 µL of each amplicon solute was transferred to a PCR tube for subsequent dual-index PCR. The index PCR assays (each 50 µL) contained 5 µL each of forward and reverse Nextera Index primers (i5 and i7) and 1X KAPA HiFi HotStart ReadyMix (Kapa Biosystems, Wilmington, MA, USA). Cycling conditions were 95°C for 3 min followed by eight cycles of 95°C for 30 s, 55°C for 30 s, and 72°C for 30 s and a final extension step of 72°C for 5 min. After the second PCR, PCR cleanup was performed with AMPure XP beads (Beckman Coulter, Brea, CA, USA) and eluted in 25 µL of TE buffer (Qiagen Cat No./ID: 19086). This TE buffer was used for all the dilution purposes throughout the process. 16S rRNA metagenomic libraries were quantified with KAPA Library Quantification Kit from Roche (catalog #07960255001, kit code KK4844), and all the libraries were normalized to a concentration of 7 nM, and then 5 µL of each sample was used to make a pool. All the first PCR and second PCR steps were followed as mentioned in the 16S Metagenomic Sequencing Library Preparation guide from Illumina. Samples were pooled in a single tube for sequencing at the University of Mississippi Medical Centre (UMMC, Jackson, MS, USA) Genomics Core Facility. Three biological replicates of each of the controls included (1) DNA extraction blank, (2) buffer, (3) sterile water, (4) no template control, and (5) positive DNA extraction control (commercially available Mock Microbial Community Standard, ZymoBIOMICS catalog #D6306). These controls were also processed during library preparation (amplicon PCR and indexing PCR) and sequencing.

### Data Processing and Analysis

Demultiplexed paired-end fastq files were provided by the UMMC. Quantitative Insights Into Microbial Ecology (QIIME2, https://qiime2.org) was used for sequence analysis. A metadata file and manifest file were created. The created metadata file was validated by Keemei (Rideout et al., 2016). The QIIME2 tutorial was followed to make the manifest file (Bolyen et al., 2019). Manifest file maps sample identifiers to fastq.gz absolute file paths that contain sequence and quality data for the sample. The manifest file is a tab-separated text file. In brief, the first column defined the sample ID, and the second column defined the absolute path to the paired-end read (forward and reverse). Manifest file is compatible to sample metadata; therefore, it could be reused to bootstrap sample metadata. “Atacama soil microbiome” tutorial and “moving pictures tutorial” were followed to process the sequencing data. Briefly, Deblur (Bokulich et al., 2013) was used for trimming, primer sequence removal, sequence denoising, paired-end merging, filtering of chimeric sequences, singleton removal, and sequence dereplication. Minimum overlap of 40 bases was used for paired-end merging. Resultant sequence sets obtained after Deblur processing were aligned by MAAFT (ver.7) (Katoh and Standley, 2013), and then a phylogenetic tree was created by using FastTree (version 2.1) (Price et al., 2010). Silva_132_99% Operational Taxonomic Unit (OTU) database (Quast et al., 2013) was used to train the naive Bayes classifier. Raw sequences were submitted to the NCBI read under SRA database and obtained accession #PRJNA728711 and PRJNA751548.

Taxonomy and metadata tables were exported from QIIME2 and uploaded as input files to the Microbiome Analyst web-based interface for visualization of bacterial relative abundances and correlation network analysis as previously described (Dhariwal et al., 2017; Chong et al., 2020). A minimum count of 10 and minimum prevalence of 20% were applied to remove low count filter from the uploaded OTU table, and a 10% interquartile range was used as the minimum for removing low variance filter features from the OTU table. Network correlation maps were inferred based on the Sparse Correlations for Compositional data (SparCC) approach (Friedman and Alm, 2012). This approach uses the log-transformed values to carry out multiple iterations, which subsequently identifies taxa that are outliers to the correlation parameters (Chong et al., 2020).

### Statistical Analysis

In our analyses, each sample was rarefied to 2,700 sequences, and the rarefied feature table was used for rarefaction analysis. Alpha diversity and beta diversity were measured by using different metrics. To compare significance in the bacterial diversity across different tick life stages and tissues, we explored the community richness and evenness using Faith_pd and Pielou_e/evenness metrics, respectively, as alpha diversity measures. The alpha-diversity group significance command creates box plots of the alpha-diversity values, and significant differences between groups are assessed with the Kruskal–Wallis test. The beta-diversity command uses box plots to visualize the distance between samples aggregated by groups specified in the metadata table file. Significant differences are assessed using a **per**mutational **m**ultiple **an**alysis **o**f **va**riance (PERMANOVA) analysis. Faith’s phylogenetic diversity (faith_pd) is an unweighted measure of alpha diversity based on phylogenetic distance in a particular sample, whereas Pielou’s evenness measures how evenly species are distributed in a sample. Kruskal–Wallis nonparametric tests (*p* ≤ 0.05) were performed to determine statistical significance of alpha-diversity metrics by using QIIME 2. Weighted and unweighted UniFrac Metrics (Lozupone and Knight, 2005) were used for beta-diversity analysis. Weighted and unweighted UniFrac metrics (Lozupone and Knight, 2005) determined the beta diversity of the whole tick life stage and tissue samples. EMPeror (Vázquez-Baeza et al., 2013) was used to create the principal component analysis (PCoA) plot, and PERMANOVA tests (*p* ≤ 0.05) were used to test the statistical significance of beta-diversity measurements. The bacterial community profile as a function of the beta diversity in both field-collected and laboratory-raised ticks was explored using the PCoA plot.

### Relative Quantification of CLE and FLE in Field-Collected Ticks From Delaware

Relative expression of CLE and FLE was estimated in UF and PF tick tissues (SG, gut, and ovary) by using quantitative reverse transcription (qRT)–PCR (**Figure 10**). Actin was used as a housekeeping gene (Additional File 2). Because of limitations of the amount of DNA in the sample, three biological replicates and two technical replicates were used during qRT-PCR assay. Briefly, 64 ng of good-quality DNA (260/280~2.0) was used for each technical replicate. Primers used in this study have been provided in Table S5. The qRT-PCR reaction mixture comprised 350 nM of each primer, and iTaq™ Universal SYBR^®^ Green Supermix (catalog #1725121). Reaction mixtures were subjected to 95°C for 6 min, followed by 45 cycles of 95°C for 27 s, 50°C for 30 s, and 72°C for 35 s using the CFX96 Real Time System (BioRad Inc., Hercules, CA, USA).

## Results

### Demultiplexing of Sequences

Illumina MiSeq sequencing generated a total of 6,832,744 demultiplexed paired-end sequences. Sequence counts in tick samples had an average of 88,737 sequences for each tick sample (Additional File 3A). Rarefaction curves relating sampling depth to the number of observed OTUs approached saturation plateau after a sampling depth of approximately 2,700 sequences (Additional File 4A). For all 77 samples, including all biological replicates, the total number of OTUs obtained was 1,666. From field-collected tick tissues, we generated a total of 3,972,116 raw, demultiplexed paired-end sequences and had an overall average read of 30,092 sequences per tick. For all 116 field-collected samples, including biological replicates, the total number of OTUs obtained was 12,891 (Additional File 3B). Each biological sample was rarefied down to a sampling depth of 5,000 sequences per sample (Additional File 4B). Overall, comparing the rarefaction analysis between laboratory-raised and field-collected tick samples showed a massive difference in the number of OTUs observed at similar sampling depth, with fewer OTUs observed in laboratory-raised ticks indicating a significant difference in diversity.

### Composition of the Microbial Communities in Life Stages and Tick Tissues From Laboratory-Raised Ticks

**Figure 2:**
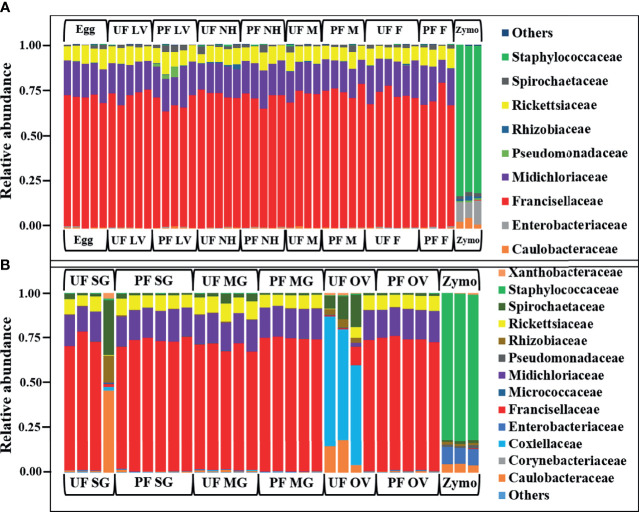
Relative abundances of bacteri profiles showing the topmost abundant bacteria families to the right of the graphs. Each bar represents a single biological replicate from different **(A)** developmental stages and **(B)** isolated tissue from laboratory raise unfed (UF) and partially fed (PF) *Amblyomma americanum* ticks. The family Francisellaceae is the most dominant group occurring at an abundance of 62% to 80% across the tick developmental stages **(A)** and 10% to 77% across isolated tissues irrespective of the blood meal. The family Coxiellaceae was detected in three of the four replicates from the unfed ovarian tissues (UF-OV) at a relative abundance of 55% to 72% **(B)**. (UF, unfed; PF, partially-fed; LV, larvae; NH, nymph; M, male; FM, female; SG, salivary gland; MG, midgut; OV, ovary; Zymo, positive bacteria control).

In whole tick samples, among all life stages, >98% of reads were covered by Francisellaceae, Midichloriaceae, Rickettsiaceae, and Spirochaetaceae. Still, in tick tissues (SG, MG, ovary), more than 98% reads are covered by Francisellaceae, Midichloriaceae, Rickettsiaceae, Spirochaetaceae, Coxiellaceae, Caulobacteriaceae, and Rhizobiaceae. In UF and blood-fed life stages (**Figure 2A**) and tissues (**Figure 2B**), Francisellaceae was found as the most dominant family with a coverage of ~70% to 75% reads except UF ovary (UF-OV) in which Coxiellaceae is the most dominant bacterial family. Of the four biological replicates of UF-OV, Coxiellaceae (55%–72%) was the dominant family in three, whereas the family Francisellaceae (71%) was abundant in one. The families Spirochaetaceae (7%–18%), Caulobacteriaceae (4%–18%), Rickettsiaceae (6%), Rhizobiaceae (~4%), and Francisellaceae (10%) were also detected in Coxiellaceae-positive UF ovarian tissues. Caulobacteriaceae and Rhizobiaceae are present only in the UF-SG and UF-OV. In UF-SG, the abundances of Caulobacteriaceae and Rhizobiaceae are 5.32% and 1.81%, respectively. In tick life stages, bacterial profiling is stable. In all UF and blood-fed tick life stages, the abundance of Francisellaceae varies from 70.5% to 73.5%, Midichloriaceae from 16.3% to 17.7%, Rickettsiaceae from 7.5% to 9.3%, and Spirochaetaceae from 1.08% to 1.58%. This result suggests hardly any impact of tick-blood feeding and tick-sex (male, female) on bacterial profiling at the genus level. Bacterial families present at <1% were categorized as others.

### Composition of the Microbial Communities From Field-Collected Ticks

**Figure 3:**
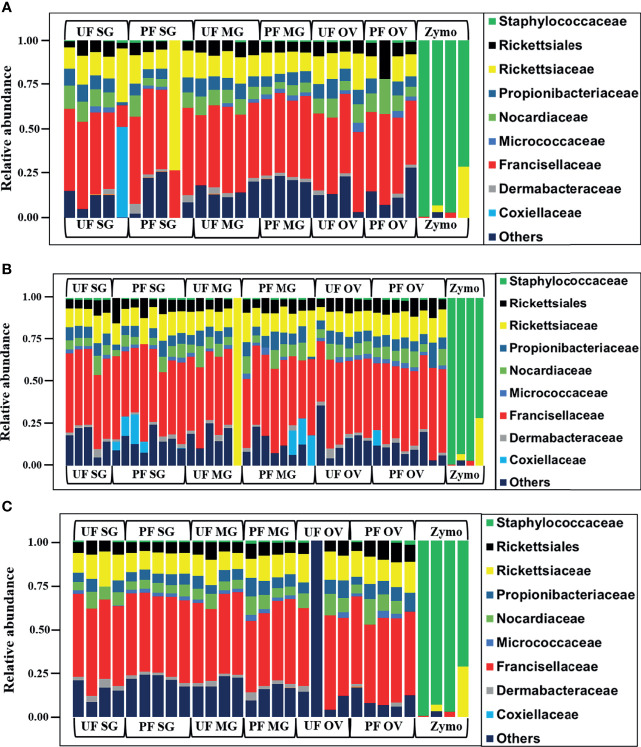
Relative abundances of bacteria profiles showing the topmost abundant bacteria families to the right of the graphs. Each column represents a single replicate from different tissues isolated from unfed (UF) and partially fed (PF) *Amblyomma americanum* ticks collected from **(A)** Illinois, **(B)** Delaware, and **(C)** Maryland. All ticks displayed a similar microbial profile with Francisellaceae being the most abundant family (26%–60%). The family Nocardiaceae, Rickettsiaceae, Propionibacteriaceae, and Rickettsiales were also present in relatively equal abundance in ticks from all three geographical locations. Three individual replicates each belonging to the partially fed salivary gland and midgut (PF-SG and PF-MG) from Delaware both have Coxiellaceae identified in their microbial composition. (UF, unfed; PF, partially fed; SG, salivary gland; MG, midgut; OV, ovary; Zymo, positive bacteria control).

We observed a similar pattern regarding bacterial taxonomy’s presence and relative abundances within tick tissues from Illinois, Delaware, and Maryland. The bacterial proportion was cataloged from the phylum down to the species level. Because of the limitation of our classifier to completely resolve identification at the genus or species level, family-level classification was used to define the patterns of bacterial assemblages identified from the field-collected ticks. The family Francisellaceae (12%–55%) was the dominant bacterial family regardless of tick tissues and geography. The only exception was the presence of Coxiellaceae (<50%) in a replicate of UF-SG and Rickettsiaceae (>70%) in a replicate of PF-SG from Illinois (**Figure 3A**), and Rickettsiaceae (100%) in a replicate of the UF-MG from Delaware (**Figure 3B**). Other predominantly identified bacterial family includes Nocardiaceae (>1%–20%), Rickettsiaceae (10%–100%), and Rickettsiales (3%–21%) (**Figures 3A–C**). An interesting observation was the presence of the family Coxiellaceae in PF-SGs (6%–17%) and PF-MG (15%–18%) from Delaware ticks (**Figure 3B**). The relative profiles of those taxa that were completely resolved to the genus and species level is attached to the supplementary data (Additional File 5).

### Diversity Analysis

#### Laboratory-Raised Ticks

**Figure 4:**
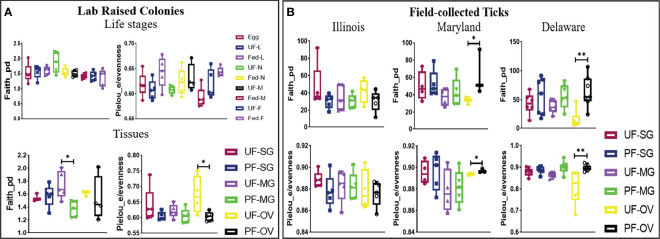
Alpha-diversity metrics. Richness (Faith_pd) and evenness (Pielou_e) index for **(A)** laboratory-raised tick tissues and developmental stages and **(B)** field collected tick tissues. Each point on the box plot represents an individual tick or tissue replicate. Asterisks designate significance level as follows **p ≤ 0.01, *p ≤ 0.05.

The alpha diversity comparison across different isolated tissues from laboratory-raised tick colonies showed significant differences in microbial richness Kruskal–Wallis: *H* = 15.87, *p* = 0.026) and evenness (Kruskal–Wallis: *H* = 17.63, *p* = 0.014) with no observed differences across developmental stages. Across different life stages from laboratory-raised tick colonies, UF-nymphs (Faith_pd value = 1.861961) and fed males (Faith_pd value = 1.40254) had the highest and lowest diversity richness index, respectively. In contrast, fed larvae (Pielou_e/evenness = 0.645623) and fed males (Pielou_e/evenness = 0.587806) showed the highest and lowest diversity evenness metric (**Figure 4A**). Among isolated tissues, UF-MG (Faith_pd value = 1.667971) and PF-MG (Faith_pd value = 1.375176) had the highest and lowest diversity richness indices, respectively. In contrast, UF-OV (Pielou_e/evenness = 0.6872987) and PF-SG (Pielou_e/evenness = 0.5937431) showed the highest and lowest diversity evenness metric (**Figure 4A**). Alpha diversity was significantly higher in UF-OVs when compared with fed ovaries (Pielou_e/evenness Kruskal–Wallis: *H* = 3.84, *p* = 0.05, *q* = 0.127) as represented in Additional File 6, whereas UF-MG showed a significantly higher alpha diversity compared with fed MG tissues based on the Faith_pd measure of microbial richness (Faith_pd Kruskal–Wallis: *H* = 5.77, *p* = 0.016, *q* = 0.19) as shown in **Figure 4A** (complete list in Additional File 7).

#### Field-Collected Ticks

The alpha diversity comparison between UF and PF tissues from field-collected ticks showed significant differences in richness (Kruskal–Wallis: *H* = 53.33, *p* = 0.005) and evenness (Kruskal–Wallis: *H* = 50.76, *p* = 0.001). Pairwise comparison of alpha diversity between UF and PF tissues showed a significant difference between the UF and PF ovarian tissues isolated from ticks from Maryland (Faith_pd Kruskal–Wallis: *H* = 3.86, *p* = 0.05, *q* = 0.200; Pielou_e/evenness: *H* = 3.86, *p* = 0.05, *q* = 0.27) and Delaware (Faith_pd Kruskal–Wallis: *H* = 7.68, *p* = 0.007, *q* = 0.12; Pielou_e/evenness: *H* = 9, *p* = 0.003, *q* = 0.21). The complete list of pairwise analysis is included as Additional Files 8 and 9). There were no observed significant differences from UF and PF tissue isolated from ticks collected from Illinois (**Figure 4B**).

**Figure 5:**
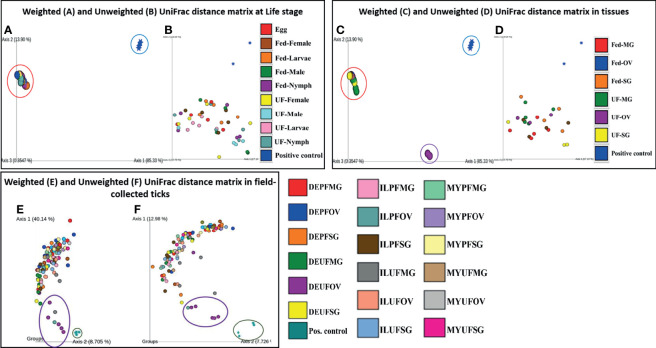
Beta diversity metrics. Emperor plot of PCoA analysis of the weighted and unweighted UniFrac distance matrix across **(A, B)** different life stage and **(C, D)** tissues of lab raised ticks. Representative PCoA analysis of **(E)** weighted and **(F)** unweighted UniFrac distance matrix from field-collected ticks. Each colored ellipse corresponds to uniquely clustering patterns of biological replicates. Each point represents the bacterial microbiome of an individual tick or dissected tissue. UF-unfed, PF-partially fed, SG-salivary gland, MG-midgut, OV-ovary, DE-Delaware, IL-Illinois, MY-Maryland.

The PCoA of the weighted (Axis 1: 85.33% and Axis 2: 13.90%) and unweighted (Axis 1: 17.10% and Axis 2: 14.64%) UniFrac distances in UF and fed life stages of laboratory-raised ticks revealed no unique clustering pattern in the bacterial communities (**Figures 5A, B**, respectively). Upon pairwise comparison, PERMANOVA indicated that few pairs of tick life-stage samples contained statistically different bacterial communities to each other, such as UF-nymph and fed-male (*p* = 0.026), or UF-nymph and UF-larvae (*p* = 0.007) (complete similarity comparison is in Additional File 10). When community profiles and similarities were explored for isolated tissues from laboratory-raised ticks, the weighted UniFrac distance matrix revealed that the UF-OV was distantly clustered compared with other tissue samples, suggesting that it contains a unique dominant bacterial community than all other tissue samples (**Figure 5C**).

**Figure 6:**
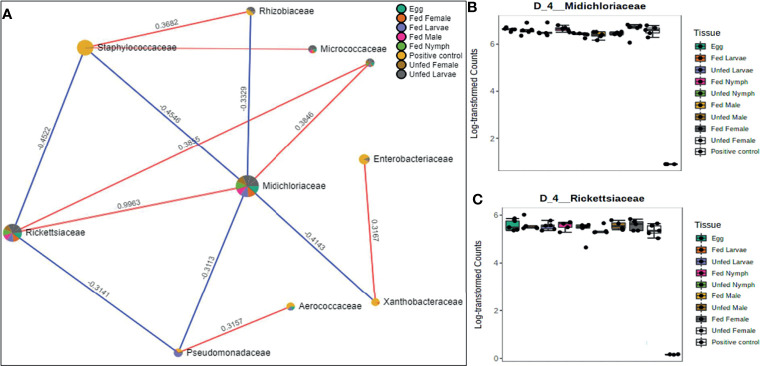
Correlation network analysis in different life stages of laboratory-raised *Amblyomma americanum* ticks. Correlation network generated using the SparCC algorithm. Correlation network with nodes representing taxa at the family level and edges representing correlations between taxa pairs **(A)** and representative box plots showing enriched abundance of Midichloriaceae **(B)**, and Rickettsiaceae **(C)** across all samples.

In contrast, the unweighted UniFrac distance matrix revealed no unique clustering pattern (**Figure 5D**). The unique clustering pattern presented by the UF-OV tissues in **Figure 6C** was further supported by the relative abundances of the bacterial families identified in this particular tissue (**Figure 2B**) and the Bray–Curtis pairwise comparison, which showed a significant difference between the composition of UF-OV tissues and fed MG tissues (Additional File 11). The dominant bacterial family in the UF-OV was Coxiellaceae (~37%), which is absent in all other tissue samples. In all other tissue samples, commonly dominant bacterial families were Francisellaceae, Midichloriaceae, Rickettsiaceae, and Spirochaetaceae, and they covered more than 90% of reads. There was no observed distinct clustering pattern in the community profiles of tick tissues isolated from field-collected ticks using both the weighted and unweighted UniFrac distance matrixes (**Figures 5E, F**). The dataset showing the pairwise comparison in field-collected ticks based on the weighted and unweighted UniFrac metrics is detailed in Additional Files 12 and 13.

### Network Correlations in the Microbiome of Laboratory-Raised Ticks

A correlation network generated using the SparCC algorithm identified 10 OTUs at the family level with 14 significant correlations across the different life stages from ticks raised in the laboratory (**Figure 6A**), of which seven were positively correlated. Of the 10 identified OTUs, 80% were not fully represented in all our samples. Only those belonging to the families Rickettsiaceae and Midichloriaceae were observed to be fully detected in all tick life stages. Interestingly, these two families (Rickettsiaceae and Midichloriaceae) were positively correlated with a significantly high correlation threshold of 0.9963 (**Figure 6A**). Rickettsiaceae and Midichloriaceae had consistently higher dominance across the tick life stages (**Figures 6B, C**).

**Figure 7:**
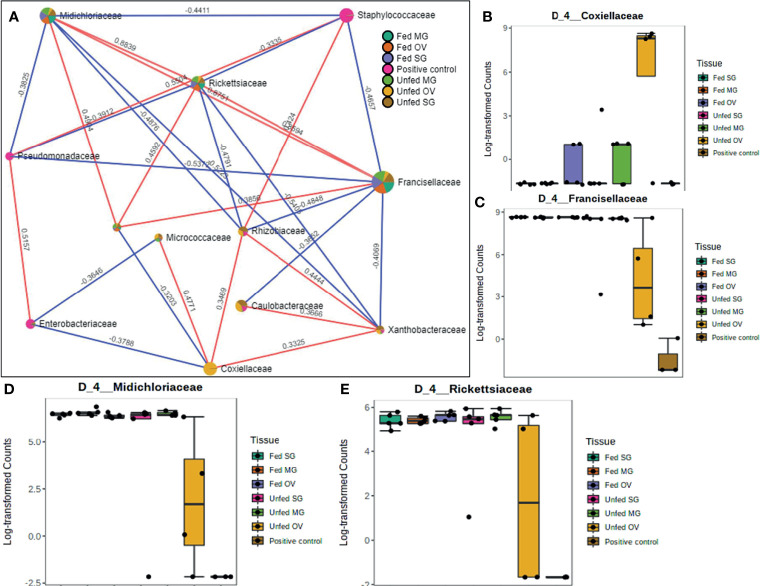
Correlation network analysis in different tissues of laboratory-raised *Amblyomma americanum* ticks. Correlation network generated using the SparCC algorithm. Correlation network with nodes representing taxa at the family level and edges representing correlations between taxa pairs **(A)** and representative boxplots showing enriched abundance of Coxiellaceae **(B)**, Francisellaceae **(C)**, Midichloriaceae **(D)**, and Rickettsiaceae **(E)** across all samples.

A total of 12 OTUs with 29 significant correlations were identified when the SparCC correlation algorithm was applied to samples representing tissues from laboratory-raised tick colonies. Approximately 48% of these correlations were positively correlated among different bacterial families, notably Francisellaceae, Rickettsiaceae, and Midichloriaceae, all of which were equally represented in all sampled tick tissues (**Figure 7A**). Coxiellaceae was dominant in UF ovarian tissues of laboratory-raised tick colonies (**Figures 2A** and **7B**) and was positively correlated with bacteria in the families Xanthobacteraceae and Rhizobiaceae, both of which showed strong negative correlation to the families Francisellaceae, Midichloriaceae, and Rickettsiaceae (**Figure 7A**), which were exclusively enriched in all other tissues except for the UF-OVs (**Figures 7C–E**). Similarly, the UF ovarian tissue exclusive Coxiellaceae was also identified to positively correlate to an unknown bacterial taxon that exhibited positive correlations to the families Francisellaceae, Rickettsiaceae, and Midichloriaceae (**Figure 7A**).

### Network Correlations in the Microbiome of Field-Collected Tick Colonies

**Figure 8:**
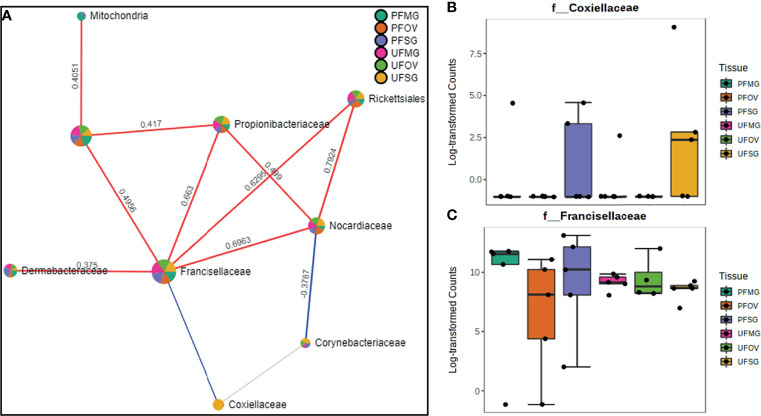
Correlation network analysis in different tissues of *Amblyomma americanum* ticks from Illinois. Correlation network generated using the SparCC algorithm. Correlation network with nodes representing taxa at the family level and edges representing correlations between taxa pairs **(A)** and representative boxplots showing enriched abundance of Coxiellaceae **(B)** and Francisellaceae **(C)** across all samples.

**Figure 9:**
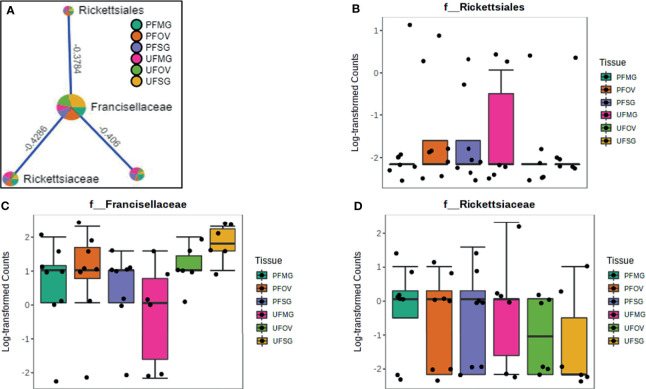
Correlation network analysis in different tissues of ticks from Delaware. Correlation network generated using the SparCC algorithm. Correlation network with nodes representing taxa at the family level and edges representing correlations between taxa pairs **(A)** and representative boxplots showing enriched abundance of Rickettsiales **(B)**, Francisellaceae **(C)**, and Rickettsiaceae **(D)** across all samples.

Our correlation analysis of field-collected tick samples only showed significant correlations within samples collected from Illinois and Delaware. We identified 12 significant correlations among nine bacterial families from ticks collected in Illinois, nine of which were positive correlations (**Figure 8A**). A negative correlation existed between the family Coxiellaceae enriched in UF-SG tissues (**Figure 8B**) and Francisellaceae, which was present across all samples (**Figure 8C**). Only four bacterial families were significantly correlated among ticks collected in Delaware (**Figures 9A–D**), and all were negative correlations.

### Relative Quantification of FLE and CLE in Field-Collected Tick Tissues

**Figure 10:**
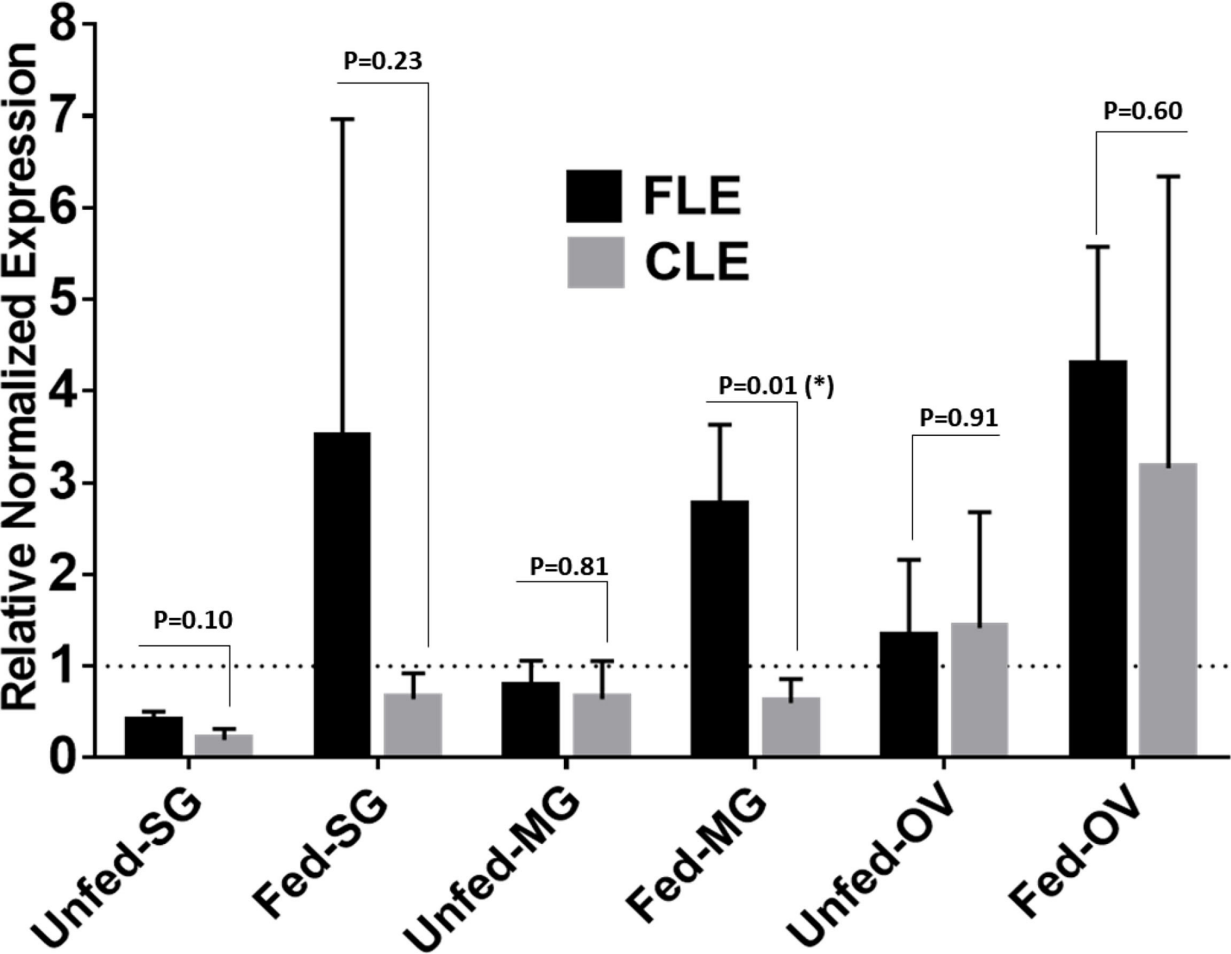
qPCR-based relative quantification of FLE and CLE in field-collected tick tissues (salivary gland, midgut and ovary) from Delaware. All the expressions shown are relative to actin. Expression of actin has been shown as 1 (dotted line). Statistically significant comparisons are indicated by *p ≤ 0.05). SG, salivary gland; MG, midgut; OV, ovary.

Relative quantification of FLE was found higher than CLE in tick tissues from field-collected *Am. Americanum* from Delaware (**Figure 10**). The relative load of FLE were found to be higher in tick tissues except for UF-OV, in which FLE and CLE levels are nearly equal (**Figure 10**). Compared with 16S data, interestingly, our qPCR data detected the expression of CLE in UF-SGs, UF-MG, and PF ovarian tissues, whereas they were not detected in the 16S data. The relative load of FLE was higher in all fed tissues compared with UF tissues with only fed MG showing significant differences (**Figure 10**).

## Discussion

The consistent occurrence of *Francisella* in both our laboratory-grown and field-collected tick samples suggests its systemic association with ticks. Our analysis has assigned abundant sequences to *Francisella* (70%–75%), followed by *Midichloria* (14%–17%) and *Rickettsia* (6%–9%), excluding UF-OV tissue. The richness of *Francisella* in immature and mature life developmental stages and individually dissected tissues (except for the UF-OV) supports the notion that it sustains an obligatory association with all feeding stages and outcompetes other bacteria. The relative abundance of *Midichloria and Rickettsia* was considerably lower than *Francisella*, signifying their link with the tick as facultative endosymbionts. However, competitive interactions among endosymbionts, pathogenic microbes, have been shown to increase virulence of pathogenic microbes, and complications in vertical transmission were suggested to influence obligatory or facultative endosymbionts in ticks (Mira and Moran, 2002). Consistent dominance of *Francisella* in tick life stages and tissues suggests its significance in *Am. americanum* development and other biological processes. Perhaps the *Am. americanum* ticks are sheltering *Francisella* by creating a favorable niche within various tissues vector for the colonization, propagation, and trafficking of *Francisella* at the expense of other endosymbionts and microbial communities. The tick vector sequestered vital nutrients from *Francisella* for development and in return provided the niche for *Francisella* to survive and thrive within the tick vector. Synergistic and antagonistic interactions among pathogenic and nonpathogenic rickettsial species could also play an important role in the prevalence of microbes within the tick vector. The interspecies competition among *Midichloria* and *Rickettsial* species could also cause their decreased prevalence within the arthropod vector. For example, dominant *R. peacockii* blocked vertical transmission of *R. rickettsii* in the ovary of *D. andersoni* (Burgdorfer et al., 1981; Niebylski et al., 1997). The dominance of FLEs in *Am. americanum* might be location-specific, as demonstrated for *Coxiella* and *Rickettsia* (Institute of Medicine (US) Committee on Lyme Disease and Other Tick-Borne Diseases, 2011; Ponnusamy et al., 2014) and of fundamental importance because of its occurrence in several other tick species such as *Dermacentor occidentalis*; *Dermacentor albipictus* (Packard); *Dermacentor nitens*; *D. andersoni*; *Dermacentor hunteri*; and *Dermacentor variabilis* (Niebylski et al., 1996; Sun et al., 2000; Scoles, 2004; Institute of Medicine (US) Committee on Lyme Disease and Other Tick-Borne Diseases, 2011; Ponnusamy et al., 2014). Still, the nature of their symbiotic relationship is yet to be explained. In this study, field-collected tick tissues from Illinois, Delaware, and Maryland also demonstrated the presence and dominance of FLEs, suggesting their establishment in field populations of *Am. americanum* ticks, although their presence seems much prominent in laboratory-grown colonies (**Figure 10**).

We found differences in the diversity metrics between laboratory-raised and field-collected ticks colonies. The observed_OTUs metrics, a quantitative measure of the total number of bacteria, showed that field-collected ticks have approximately 100 folds more bacteria than laboratory-raised ticks irrespective of sample type (**Figure S1**). We also found that field-collected ticks have a higher diverse microbiome regardless of the tick location than laboratory-raised tick colonies. To further strengthen this observation, despite the fact Francisellaceae was the dominant bacterial family in both tick groups, field-collected ticks also harbored other bacterial families at a relatively high abundance contributing to a more diverse microbiome. This finding implies that the tick environment plays a significant role in enriching certain bacteria members of the tick microbiome. The laboratory-raised ticks have been maintained under strict laboratory conditions with limited vertebrate hosts and have less richness in their microbial communities. It seems possible that the differences in the diversity observed among life stages of field-collected and laboratory-raised ticks are due solely to the continuous exposure to environmental bacteria encountered by questing ticks in the field. A closer look at bacterial profile in **Figures 2** and **3** clearly indicates that with the exception of the presence of Francisellaceae in both laboratory-raised and field-collected ticks, several bacterial family were exclusively detected in field-collected ticks such as Propionibacteriaceae, Nocardiaceae, Dermabacteraceae, Rickettsiales, and Staphylococcaceae, all of which are known to be environmental residents in soils and are predominant on both human and animal skin. These findings provide further support for the hypothesis that the tick microbiome may play a contributing factor to the incidence of the alpha-gal phenomenon across different region in the United States.

An earlier study conducted on field-collected *Am. americanum* ticks from Indiana showed approximately 40% of CLEs, followed by ~5% rickettsial endosymbionts (Institute of Medicine (US) Committee on Lyme Disease and Other Tick-Borne Diseases, 2011). A recent microbiome study of *Am. americanum* from Kansas reported a dominance of endosymbiotic genera belonging to *Coxiella* and *Rickettsia* (>97%) (Maldonado-Ruiz et al., 2021). However, ticks collected from North Carolina harbored a preponderance of *Rickettsia*-like endosymbionts over *Coxiella* (Ponnusamy et al., 2014). In the current study, the most abundant bacterial family is *Francisella* at the organismal level in both immature and adult tick life stages and individually dissected tissues (excluding UF-OV), suggesting that the microbiome composition of this tick varies with geographical distribution. The presence of *Coxiella* in UF-OV tissue indicates its obligate symbiont nature, and another study has shown its presence in the ovary (to show its maternal transmission) and Malpighian tubules to suggest its possible role in osmoregulation and excretion (Klyachko et al., 2007; Machado-Ferreira et al., 2011; Lalzar et al., 2014). Surprisingly, CLE was detected in neither tick developmental life stages nor tissue samples, except in the UF-OV. CLE, an obligatory endosymbiont, is required for tick survival and reproductive fitness (Zhong et al., 2007). CLE has been implicated for its possible role in synthesizing amino acids and several B vitamins (Klyachko et al., 2007; Machado-Ferreira et al., 2011; Lalzar et al., 2014). Possible reasons for the replacement of CLE in *Am. americanum* could be the acquisition of FLE and Rickettsiae established as alternative obligate symbionts in some tick species (Duron et al., 2017). All genes required for vitamin B_9_ and B_7_ biosynthesis are also present in *Rickettsia* and FLE endosymbionts, respectively. In instances where multiple endosymbionts provide a similar advantage to the host, the presence and maintenance of all endosymbionts are not expected; it does not add additional help to the tick host (Vautrin and Vavre, 2009).

It was a common understanding that hematophagous niches are commonly driven by evolutionarily stable symbiotic interactions in several arthropods (Moran et al., 2008; Wernegreen, 2012). Contrary to that, an elegant study proposed that obligate symbioses are relatively unstable in obligate hematophagous ticks (Duron et al., 2017). In that study, six genera of distinct endosymbionts were present in the castor bean tick (*Ixodes ricinus)* and the African blue tick (*Rhipicephalus decoloratus*), but no symbiotic community structure was found fixed and stable across the tick phylogeny (Duron et al., 2017). The success of horizontal and vertical transmission patterns in ticks could also modify symbiotic interactions (Duron et al., 2017). This fact could also explain why there is low evolutionary stability of the symbiosis between *Am. americanum* ticks and CLE, or CLEs are missing from most of our tick samples in the current study.

CLE follows two different evolutionary strategies. Some CLEs are highly specialized to the tick host from ancient times, followed by codiversification. For example, the *Rhipicephalus* genus and CLE lineages have codiversified together. The emergence of the *Rhipicephalus* genus and original CLE infection co-occurred, approximately 14 million years ago (Murrell et al., 2001; Duron et al., 2017; Ben-Yosef et al., 2020). On the other hand, other CLEs appear to be acquired through HT from unrelated or accidental host species. Such a pattern has also been observed in other endosymbionts such as *Wolbachia*, which occurs frequently in insects, similar to CLE in ticks. A recent study also supports the hypothesis of frequent replacement of obligate symbiont CLE in ticks (Duron et al., 2017). Our data provide strong evidence that CLE replacement by FLE occurs in *Am. americanum* populations; otherwise, FLEs are rare in ticks and not frequently found in arthropods (Niebylski et al., 1997; Scoles, 2004; Goethert and Telford, 2005; Clayton et al., 2015; Gerhart et al., 2016; Duron et al., 2017).

Earlier studies proposed that among *Amblyomma* tick species, CLEs have reduced genomes, a feature associated with microbes that are long-term or early evolved endosymbionts (McCutcheon and Moran, 2011). A wide distribution of genetically differentiated strains of CLE across the tick phylogeny also specifies its ancient symbiotic relation with ticks (Duron et al., 2017). Unlike the highly reduced genome of CLEs, FLEs have minimal genome reduction and evolved recently from a mammalian pathogen *F. tularensis* (Gerhart et al., 2016) and have more superior biosynthetic metabolic capabilities than CLEs. Therefore, it is highly likely that it has replaced ancestral CLEs with reduced functionality in *Am. americanum* developmental stages and tissue samples, as evident from the data presented here. A recent study has also suggested replacement of endosymbiont CLEs with FLEs in another *Amblyomma* tick species, the Gulf Coast tick (*Amblyomma maculatum*), for the same reason (Gerhart et al., 2016). It is also possible that *Am. americanum* replaced CLE at the expense of functionally important symbiotic genes (via lateral gene transfer), with FLEs that are functionally more efficient than CLEs because CLE has a reduced genome and is less efficient in metabolic and biosynthetic capabilities, including that of B vitamin synthesis. Perhaps, this is the key evolutionary mechanism of how ticks retain their capacity to synthesize vitamin B even without containing endosymbionts. Previously, this pattern has been reported from some filarial nematodes, which demonstrated the ability to live and reproduce without obligate symbionts through lateral gene transfer (McNulty et al., 2010). A close examination of *I. scapularis*, a tick species deficient in CLE, did not show any sign or evidence of lateral gene transfer (Gulia-Nuss et al., 2016). However, it contained a rickettsial endosymbiont, *Rickettsia buchneri*, which could synthesize the B_9_ vitamin (Hunter et al., 2015). The presence of *R. buchneri* provides a possible explanation for both the absence of CLE and no evidence of lateral gene transfer. An unexplored ecological pathway area facilitating the HT of endosymbionts among tick species needs to be thoroughly investigated. Cofeeding of different tick species on a shared vertebrate host could be an important determinant. Reports have also shown that the SGs of blood-fed ticks contain high levels of CLE in some tick species (Klyachko et al., 2007; Qiu et al., 2014). The geographic expansion of *Am. americanum* in new territories, including the southern coastal areas along with the Atlantic coast and the Gulf of Mexico and several northern states in the United States, has been reported and predicted (Molaei et al., 2019; Raghavan et al., 2019). The lone star tick (*Am. americanum*) and American dog tick (*D. variabilis*) are historically prevalent in these areas and naturally harbor FLEs (Budachettri et al., 2014; Travanty et al., 2019). A variety of reservoir hosts play an essential role in the maintenance of these endosymbionts. For example, wild rabbits, hare, and muskrats are known reservoirs for *Francisella* species (Nigrovic and Wingerter, 2008). Hence, a change in host feeding due to new expansion in these areas could be one reason for establishing FLEs in *Am. americanum.* As cofeeding is reported as one significant factor for exchanging virome among *D. variabilis*, *Am. americanum*, and *I. scapularis* (Tokarz et al., 2014). Thus, the cofeeding of tick species on a shared vertebrate host could serve as an ecological arena for exchanging endosymbionts. Earlier studies have suggested that tick pathogens also colonize with closely related endosymbionts, and these endosymbionts also appear to promote closely related pathogen acquisition and transmission (Bonnet et al., 2017). The FLEs are known to have evolved from the pathogen *F. tularensis* (Ft); however, unlike virulent Ft, its transmission and virulence in humans are enigmas (Ivanov et al., 2011; Rounds et al., 2012; Wójcik-Fatla et al., 2015). A wide distribution of FLEs in other tick species has been further highlighted by the reports such as >94% of FLE-positives among *D. variabilis*, *D. andersoni*, and *D. occidentalis* ticks in the Western United States (Niebylski et al., 1997; Rounds et al., 2012) and 41% FLE-positives in *D. occidentalis* ticks from California (Western United States) but without Ft infection (Gurfield et al., 2017). In another earlier study, *D. andersoni* ticks collected from Oregon and Montana (northwestern United States) showed FLE and Ft accounted for 80% (60% FLEs and 20% Ft) of MG microbiome (Gall et al., 2016), suggesting that genetic similarity of FLE and Ft and geography are both probably contributing to inflating Ft infection rates. More studies are needed to clarify the involvement of FLEs in Ft infection and how factors such as geography and genetic similarity are involved.

Our network analysis showed a strong positive correlation between Rickettsiaceae and Midichloriaceae families in laboratory-raised tick life stages and between Francisellaceae, Rickettsiaceae, and Midichloriaceae in tissues isolated from laboratory-raised ticks. Interestingly, the presence of the Rickettsiaceae family was shown to be positively correlated to Midichloriaceae across different life stages of laboratory-raised tick colonies, indicative of a potential synergistic relationship between bacteria belonging to these two families. This observation was also reported in another study (Budachetri et al., 2018). It showed the *Rickettsia parkeri* colonization of the tick tissue facilitates replication of the endosymbiont *Candidatus* Midichloria *mitochondrii* (CMM) in *Am. maculatum* ticks. Lejal et al. (2021) also identified a substantial prevalence of CMM in *I. ricinus* ticks that were positive with bacteria in the *Rickettsia* genus.

Coxiellaceae is replaced with Francisellaceae in *Am. americanum* ticks, and this pattern was further strengthened by analyzing the correlations between bacterial families identified in tissues of laboratory-raised tick colonies. While we observed no direct correlation among bacteria in the Coxiellaceae family and Rickettsiaceae, Francisellaceae, and Midichloriaceae, the Coxiellaceae family was positively correlated with Rhizobiaceae and Xanthobacteraceae, both of which were negatively correlated to Rickettsiaceae, Francisellaceae, and Midichloriaceae bacterial families. Both Rhizobiaceae and Xanthobacteraceae are nonresident, opportunistic bacteria that ticks acquire from their environment (Sun et al., 2000; Scoles, 2004; Andreotti et al., 2011; Carpi et al., 2011; Institute of Medicine (US) Committee on Lyme Disease and Other Tick-Borne Diseases, 2011; Ponnusamy et al., 2014; Clayton et al., 2015; Galili, 2016; Gurfield et al., 2017). Lejal and colleagues (2021) detected a significant abundance of a Rhizobiaceae-Multi_1 in *Rickettsia-*positiv*e I. ricinus* ticks, suggesting a completely different observation of our findings that Rhizobiaceae was negatively correlated with the Rickettsiaceae, but positively correlated with Coxiellaceae. This finding could present a potential interaction between known bacterial endosymbionts and possible environmental bacteria transiently acquired by ticks. An understanding of how these environmental microbes change the dynamics of the tick microbiome requires further attention.

The presence of specific microbes in tick SGs is vital in the context of AGS as tick bites are believed to be responsible for causing AGS in humans. The lone star tick possesses alpha-gal antigens in its SG and saliva, which are hypothesized to be prime culprits of AGS (Crispell et al., 2019). The gene that codes for the enzyme α-1,3-galactosyltransferase has not been identified in a tick so far, indicating that the microbiome of the tick SG could be one major contributor of alpha-gal antigens (Sharma and Karim, 2021; Sharma et al., 2021). Several pieces of evidence support this hypothesis. One important tick-borne bacterial pathogen, *Anaplasma phagocytophilum*, is linked with an increase of alpha-gal antigen (Cabezas-Cruz et al., 2019). Similarly, *Francisella* species are also reported to be involved in modulating glycosylation genes such as glycosyltransferase and glycosidase in the host (Chacko et al., 2011). Hence, it can be speculated that the establishment of FLE can contribute toward the rise of alpha-gal signatures in ticks. The recent change of guard within *Am. americanum* (CLE replaced by FLE) points to the potential role of the tick microbiome in the emergence of AGS. The exclusive replacement of the *Coxiellaceae* with *Francisellaceae* in both laboratory-raised and field-collected *Am. americanum* ticks could shed more light on the role played by this tick in the emergence of AGS. Although there are no current studies on when the pathogenic *F. tularensis* switches to an endosymbiont, Gerhart et al. (2016) argued that an FLE of *Am. maculatum* recently evolved to becoming an endosymbiont. Similarly, it is worth noting that the emergence of AGS in humans and the incrimination of *Am. americanum* in inducing this condition are recent development. Understanding whether these two phenomena took place concurrently or simultaneously will significantly fill a huge gap in our understanding of the microbiome–tick vector interaction in the context of AGS.

An elegant recent study conducted on the human gut microbiome reported gut resident microbes from the Enterobacteriaceae family, including *Escherichia coli*, *Pasteurellaceae* genera, *Haemophilus influenzae*, and *Lactobacillus* species, can exhibit α-1,3-galactosyltransferase activity, which indicates that the presence of an enzyme in these microbes could contribute to the devolvement of an alpha-gal antigen (Montassier et al., 2020). This study also identified specific genes exhibiting α-1,3-galactosyltransferase bacterial sequences in their shotgun sequencing data (Montassier et al., 2020). An earlier study reported microbes from Rhizobiaceae and Caulobacteriaceae families possessing novel lipid A a-(1,1)-GalA transferase gene(*rgtF*) (Brown et al., 2013). This enzyme could also be a potential source of an α- gal antigen. In this study, we identified microbes from Rhizobiaceae and Caulobacteriaceae family in *Am. americanum* SG samples. The microbes from these families could contribute to the synthesis of an alpha-gal antigen or overall alpha-gal signature in tick SGs. The tick microbiome possessing an uncharacterized enzyme with a glycoside hydrolase, glycosyltransferase, or similar function as α-1,3-galactosyltransferase, is yet to be investigated.

## Conclusion

In this study, a detailed and comprehensive 16S rRNA sequencing revealed a stable microbiome composition in all developmental stages of the lone star tick (*Am. americanum*), and the traditionally associated CLE was absent in these developmental stages (excluding the UF-OV), thus pointing to a solid niche for vertical transmission to the next generation. Interestingly, the *Am. americanum* ticks investigated appear to have acquired another tick endosymbiont, FLE, at the expense of CLE. Levels of FLE were significantly higher in laboratory-maintained ticks than field-collected ticks, possibly due to exposure to limited host species used for tick rearing compared with more diverse vertebrate hosts in the wild and/or controlled laboratory conditions compared with field environmental conditions. Historically, CLE is known to benefit its tick host, synthesizing vitamin B and cofactors that aid in osmoregulation, excretion, and reproduction. The possible rationale for acquiring and establishing FLE at the expense of CLE could be the reduced genome of CLE leading to impaired vitamin B synthesis when compared with FLE. FLE coverage in tick life stages was ~70% of total reads, suggesting its significance in tick physiology. An inverse relationship between the number of FLEs and spotted fever group Rickettsia (SFGR) has been demonstrated in *D. occidentalis*, suggesting that FLEs can interfere with SFGR colonization. It has also been shown that a high abundance of FLEs is positively correlated with the acquisition of pathogen *Francisella novicida*. Could this positive correlation between FLE and pathogen *F. novicida* lead to a possible infection of *F. novicida* in lone star ticks in the east-central United States soon?

## Data Availability Statement

The datasets presented in this study can be found in online repositories. The names of the repository/repositories and accession number(s) can be found in the article/**Supplementary Material**.

## Ethics Statement

The animal study was reviewed and approved by University of Southern Mississippi IACUC.

## Author Contributions

SK conceptualized the study. DK, SS, AA, and SK were responsible for data curation and formal analysis. Funding acquisition was done by SK. Investigation was performed by SS, DK, AA, and SK. SS, DK, AA, AK, HT, AL, and SK were responsible for the methodology of the study. SK was responsible for project administration, resources, and supervision. Validation was performed by DK, SS, and SK. Visualization was performed by SS, DK, AA, and SK. DK, SS, AA, and SK were responsible for writing of the original draft. DK, SS, AA, AK, HT, AL, and SK were responsible for writing, review, and editing. All authors contributed to the article and approved the submitted version.

## Funding

This research was principally supported by USDA NIFA award #2017-67017-26171; the National Institutes of Allergy and Infectious Diseases award, RO1 AI135049; Pakistan-US Science and Technology Cooperation award (US Department of State); the Mississippi INBRE [an institutional Award (IDeA) from the National Institute of General Medical Sciences of the National Institutes of Health under award P20GM103476]. The funders played no role in the study design, data collection and analysis, decision to publish, or preparation of the manuscript.

## Conflict of Interest

The authors declare that the research was conducted in the absence of any commercial or financial relationships that could be construed as a potential conflict of interest.

## Publisher’s Note

All claims expressed in this article are solely those of the authors and do not necessarily represent those of their affiliated organizations, or those of the publisher, the editors and the reviewers. Any product that may be evaluated in this article, or claim that may be made by its manufacturer, is not guaranteed or endorsed by the publisher.

## Abbreviations

*Am, americanum*, *Amblyomma americanum*; PCoA, Principal component analysis; OUT, Operational taxanomic unit; TBD, Tick-borne disease; TBP, Tick-borne pathogens; FLE, *Francisella*-like endosymbiont; CLE, *Coxiella*-like endosymbiont; AGS, Alpha-gal syndrome; QIIME2, Quantitative Insights into Microbial Ecology 2; PERMANOVA, Permutational multiple analysis of variance; SparCC, Sparse Correlations for Compositional data
