# Supplementary material for: Recently Evolved Francisella-Like Endosymbiont Outcompetes an Ancient and Evolutionarily Associated Coxiella-Like Endosymbiont in the Lone Star Tick (Amblyomma americanum) Linked to the Alpha-Gal Syndrome
Source: Front Cell Infect Microbiol. 2022 Apr 12;12:787209. doi: 10.3389/fcimb.2022.787209 (PMC9039623; doi:10.3389/fcimb.2022.787209)
Supplement: Supplementary file 1 [file DataSheet_1.docx]

SUPPLEMENTARY INFORMATION

Additional File1: PCR and index primers used for V3-V4 amplification.

|  | 1st PCR primers |  |
| --- | --- | --- |
| 1st PCR primer-16S-V3-V4-Forward | 5'TCGTCGGCAGCGTCAGATGTGTATAAGAGACAGCCTACGGGNGGCWGCAG3' | Forward overhang P5-tag |
| 1st PCR primer-16S-V3-V4-Reverse | 5'GTCTCGTGGGCTCGGAGATGTGTATAAGAGACAGGACTACHVGGGTATCTAATCC3' | Locus-specific primer |
|  |  | Reverse overhang P7-tag |
| P5-PCR index primer:F | 2nd PCR primers | i5 index Name |
| F1 | 5’AATGATACGGCGACCACCGAGATCTACAC[TAGATCGC]TCGTCGGCAGCGTC | [E/H/N/S]501 |
| F2 | 5’AATGATACGGCGACCACCGAGATCTACAC[CTCTCTAT]TCGTCGGCAGCGTC | [E/H/N/S]502 |
| F3 | 5’AATGATACGGCGACCACCGAGATCTACAC[TATCCTCT]TCGTCGGCAGCGTC | [E/H/N/S]503 |
| F4 | 5’AATGATACGGCGACCACCGAGATCTACAC[AGAGTAGA]TCGTCGGCAGCGTC | [E/H/N/S]504 |
| F5 | 5’AATGATACGGCGACCACCGAGATCTACAC[GTAAGGAG]TCGTCGGCAGCGTC | [E/H/N/S]505 |
| F6 | 5’AATGATACGGCGACCACCGAGATCTACAC[ACTGCATA]TCGTCGGCAGCGTC | [E/H/N/S]506 |
| F7 | 5’AATGATACGGCGACCACCGAGATCTACAC[AAGGAGTA]TCGTCGGCAGCGTC | [E/H/N/S]507 |
| F8 | 5’AATGATACGGCGACCACCGAGATCTACAC[CTAAGCCT]TCGTCGGCAGCGTC | [E/H/N/S]508 |
| F9 | 5’AATGATACGGCGACCACCGAGATCTACAC[CGTCTAAT]TCGTCGGCAGCGTC | [E/H/N/S]510 |
| F10 | 5’AATGATACGGCGACCACCGAGATCTACAC[TCTCTCCG]TCGTCGGCAGCGTC | [E/H/N/S]511 |
| F11 | 5’AATGATACGGCGACCACCGAGATCTACAC[TCGACTAG]TCGTCGGCAGCGTC | [E/H/N/S]513 |
| F12 | 5’AATGATACGGCGACCACCGAGATCTACAC[TTCTAGCT]TCGTCGGCAGCGTC | [E/H/N/S]515 |
| F13 | 5’AATGATACGGCGACCACCGAGATCTACAC[CCTAGAGT]TCGTCGGCAGCGTC | [E/H/N/S]516 |
| F14 | 5’AATGATACGGCGACCACCGAGATCTACAC[GCGTAAGA]TCGTCGGCAGCGTC | [E/H/N/S]517 |
| F15 | 5’AATGATACGGCGACCACCGAGATCTACAC[CTATTAAG]TCGTCGGCAGCGTC | [E/H/N/S]518 |
| F16 | 5’AATGATACGGCGACCACCGAGATCTACAC[AAGGCTAT]TCGTCGGCAGCGTC | [E/H/N/S]520 |
| F17 | 5’AATGATACGGCGACCACCGAGATCTACAC[GAGCCTTA]TCGTCGGCAGCGTC | [E/H/N/S]521 |
| F18 | 5’AATGATACGGCGACCACCGAGATCTACAC[TTATGCGA]TCGTCGGCAGCGTC | [E/H/N/S]522 |

| P7-PCR index primer:R |  | i7 bases on sample sheet | i7 index name |
| --- | --- | --- | --- |
| R1 | 5’CAAGCAGAAGACGGCATACGAGAT[TCGCCTTA]GTCTCGTGGGCTCGG |  | [H/N]701 |
| R2 | 5’CAAGCAGAAGACGGCATACGAGAT[CTAGTACG]GTCTCGTGGGCTCGG |  | [H/N]702 |
| R3 | 5’CAAGCAGAAGACGGCATACGAGAT[TTCTGCCT]GTCTCGTGGGCTCGG |  | [H/N]703 |
| R4 | 5’CAAGCAGAAGACGGCATACGAGAT[GCTCAGGA]GTCTCGTGGGCTCGG |  | [H/N]704 |
| R5 | 5’CAAGCAGAAGACGGCATACGAGAT[AGGAGTCC]GTCTCGTGGGCTCGG |  | [H/N]705 |
| R6 | 5’CAAGCAGAAGACGGCATACGAGAT[CATGCCTA]GTCTCGTGGGCTCGG |  | [H/N]706 |
| R7 | 5’CAAGCAGAAGACGGCATACGAGAT[GTAGAGAG]GTCTCGTGGGCTCGG |  | [H/N]707 |
| R8 | 5’CAAGCAGAAGACGGCATACGAGAT[CCTCTCTG]GTCTCGTGGGCTCGG |  | [H/N]708 |
| R9 | 5’CAAGCAGAAGACGGCATACGAGAT[AGCGTAGC]GTCTCGTGGGCTCGG |  | [H/N]709 |
| R10 | 5’CAAGCAGAAGACGGCATACGAGAT[CAGCCTCG]GTCTCGTGGGCTCGG |  | [H/N]710 |
| R11 | 5’CAAGCAGAAGACGGCATACGAGAT[TGCCTCTT]GTCTCGTGGGCTCGG |  | [H/N]711 |
| R12 | 5’CAAGCAGAAGACGGCATACGAGAT[TCCTCTAC]GTCTCGTGGGCTCGG |  | [H/N]712 |
| R13 | 5’CAAGCAGAAGACGGCATACGAGAT[TCATGAGC]GTCTCGTGGGCTCGG |  | [H/N]714 |
| R14 | 5’CAAGCAGAAGACGGCATACGAGAT[CCTGAGAT]GTCTCGTGGGCTCGG |  | [H/N]715 |
| R15 | 5’CAAGCAGAAGACGGCATACGAGAT[TAGCGAGT]GTCTCGTGGGCTCGG | ACTCGCTA | [H/N]716 |
| R16 | 5’CAAGCAGAAGACGGCATACGAGAT[GTAGCTCC]GTCTCGTGGGCTCGG | GGAGCTAC | [H/N]718 |
| R17 | 5’CAAGCAGAAGACGGCATACGAGAT[TACTACGC]GTCTCGTGGGCTCGG | GCGTAGTA | [H/N]719 |
| R18 | 5’CAAGCAGAAGACGGCATACGAGAT[AGGCTCCG]GTCTCGTGGGCTCGG | CGGAGCCT | [H/N]720 |
| R19 | 5’CAAGCAGAAGACGGCATACGAGAT[GCAGCGTA]GTCTCGTGGGCTCGG | TACGCTGC | [H/N]721 |
| R20 | 5’CAAGCAGAAGACGGCATACGAGAT[CTGCGCAT]GTCTCGTGGGCTCGG | ATGCGCAG | [H/N]722 |
| R21 | 5’CAAGCAGAAGACGGCATACGAGAT[GAGCGCTA]GTCTCGTGGGCTCGG | TAGCGCTC | [H/N]723 |
| R22 | 5’CAAGCAGAAGACGGCATACGAGAT[CGCTCAGT]GTCTCGTGGGCTCGG | ACTGAGCG | [H/N]724 |
| R23 | 5’CAAGCAGAAGACGGCATACGAGAT[GTCTTAGG]GTCTCGTGGGCTCGG | CCTAAGAC | [H/N]726 |
| R24 | 5’CAAGCAGAAGACGGCATACGAGAT[ACTGATCG]GTCTCGTGGGCTCGG | CGATCAGT | [H/N]727 |
| R25 | 5’CAAGCAGAAGACGGCATACGAGAT[TAGCTGCA]GTCTCGTGGGCTCGG | TGCAGCTA | [H/N]728 |
| R26 | 5’CAAGCAGAAGACGGCATACGAGAT[GACGTCGA]GTCTCGTGGGCTCGG | TCGACGTC | [H/N]729 |

Additional File 2: Gene-specific qRT PCR primers used in this study.

| Gene | Gene | Forward Primer (5'-3') | Reverse Primer (5'-3') | Size (bp) | Reference |
| --- | --- | --- | --- | --- | --- |
| *FLE-16S rRNA* | 16S rRNA | CTGATCCAGCAATGCCATGT | ACGTCCTTCCTCAAGGCTATTA | 100 | This study |
| *CLE-16S rRNA* | 16S rRNA | CCTTTTGAGCGTTGACGTTA | CCAAAGGCACCAAGTCATTT | 566 | [72] |
| *Aa β-Actin* | EZ000248.1 | TGGTATCCTCACCCTGAAGTA | ACGCAGCTCGTTGTAGAA | 100 | This study |

Additional File 3:

| Additional File 3: Demultiplexed sequence counts summary | | |
| --- | --- | --- |
| A) Lab-raised ticks | | |
|  | Forward reads | Reverse reads |
| Mean | 88736.9 | 88736.9 |
| Maximum | 358604 | 358604 |
| Total | 6832744 | 6832744 |
|  |  |  |
| B) Field-collected ticks | | |
|  | Forward reads | Reverse reads |
| Mean | 30091.8 | 30091.8 |
| Maximum | 177785 | 177785 |
| Total | 3972116 | 3972116 |

Additional File 4: Result of rarefaction analysis of sequences from individual samples

Figure S1. Alpha-rarefaction curve of all A. americanum biological replicates from different developmental stages and tissues. Each curve representing individual replicates was rarified to a sequence depth of approximately 2500 sequences for A) lab-raised tick tissues and developmental stages (unfed and partially fed), and 5000 sequences for B) field-collected tick tissues (unfed and partially fed).


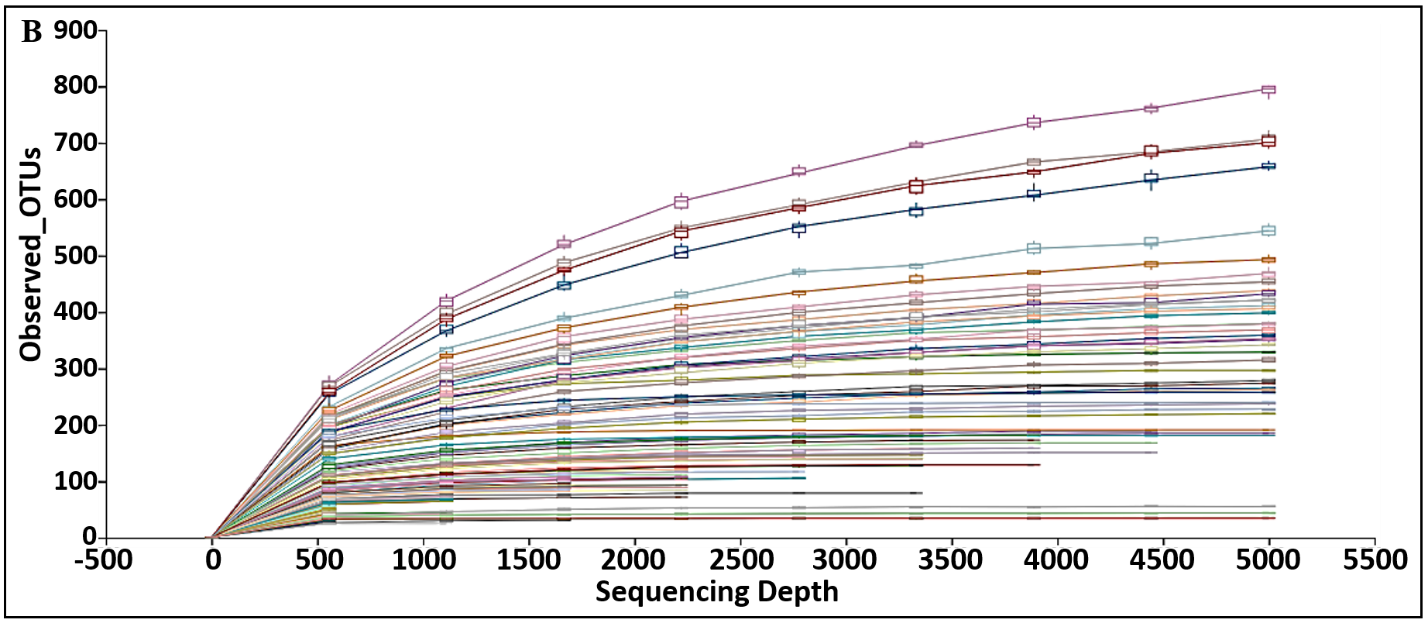

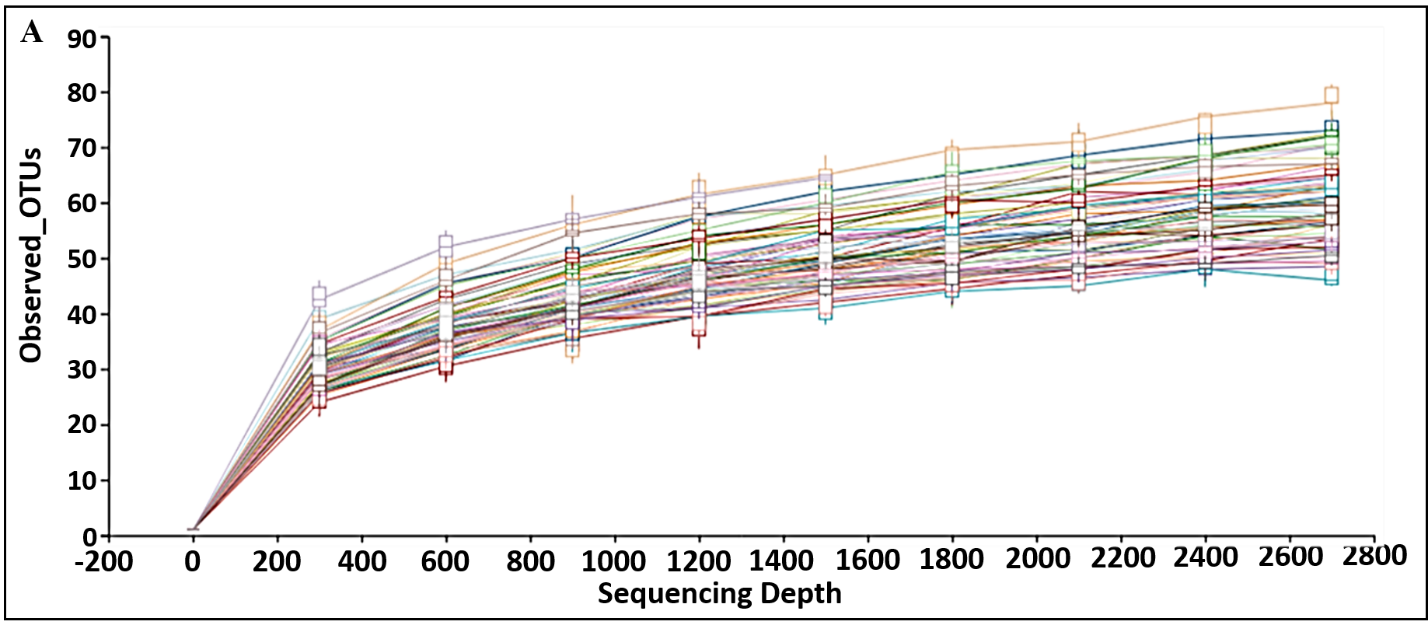


Additional File 5:

**A B**


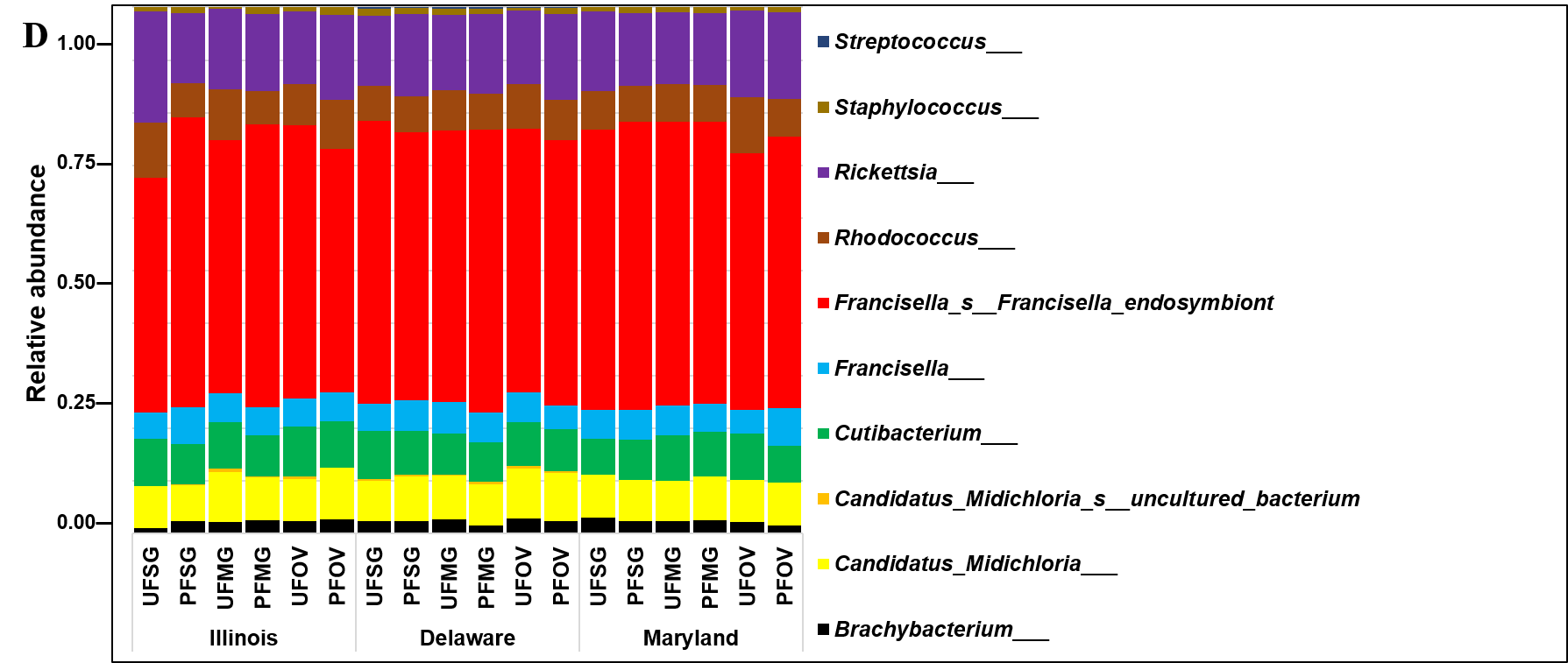

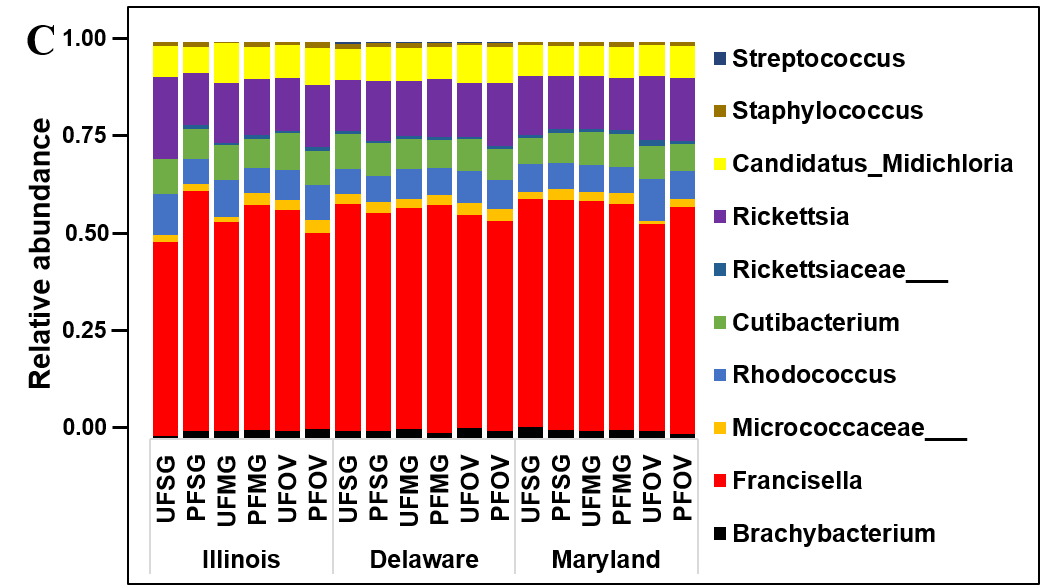

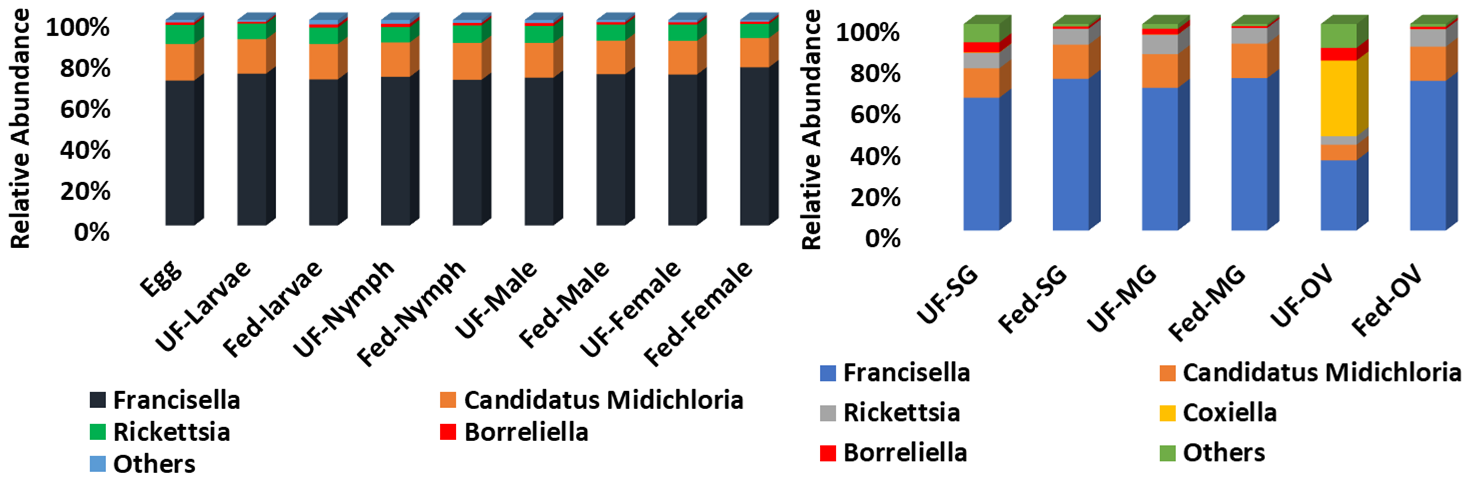


Additional File 5: Microbial diversity of *A. americanum* (Aa) in life stages (A) and tissues (B) at Genus level.

Additional File 6: Kruskal-Wallis-alpha diversity (evenness) pairwise among *Amblyomma americanum* tissue samples

| Group 1 | Group 2 | H | p-value | q-value |
| --- | --- | --- | --- | --- |
| Aa Unfed Adult Female MG (n=5) | Aa Unfed Adult Female OV (n=4) | 1.5 | 0.22067136 | 0.31893534 |
| Aa Unfed Adult Female MG (n=5) | Aa Unfed Adult Female SG (n=5) | 0.0109091 | 0.91681495 | 0.91681495 |
| Aa Unfed Adult Female MG (n=5) | Aa fed Adult Female MG (n=5) | 1.8436364 | 0.17452534 | 0.30709277 |
| Aa Unfed Adult Female MG (n=5) | Aa fed Adult Female OV (n=5) | 2.4545455 | 0.11718509 | 0.23437017 |
| Aa Unfed Adult Female MG (n=5) | Aa fed Adult Female SG (n=5) | 1.32 | 0.25059205 | 0.31893534 |
| Aa Unfed Adult Female MG (n=5) | positve control-zymo (n=3) | 5 | 0.02534732 | 0.11828749 |
| Aa Unfed Adult Female MG (n=5) | x (n=44) | 0.0392727 | 0.84290934 | 0.8741282 |
| Aa Unfed Adult Female OV (n=4) | Aa Unfed Adult Female SG (n=5) | 1.5 | 0.22067136 | 0.31893534 |
| Aa Unfed Adult Female OV (n=4) | Aa fed Adult Female MG (n=5) | 3.84 | 0.05004352 | 0.12738351 |
| Aa Unfed Adult Female OV (n=4) | Aa fed Adult Female OV (n=5) | 3.84 | 0.05004352 | 0.12738351 |
| Aa Unfed Adult Female OV (n=4) | Aa fed Adult Female SG (n=5) | 3.84 | 0.05004352 | 0.12738351 |
| Aa Unfed Adult Female OV (n=4) | positve control-zymo (n=3) | 4.5 | 0.03389485 | 0.12738351 |
| Aa Unfed Adult Female OV (n=4) | x (n=44) | 3.9086271 | 0.04803883 | 0.12738351 |
| Aa Unfed Adult Female SG (n=5) | Aa fed Adult Female MG (n=5) | 1.32 | 0.25059205 | 0.31893534 |
| Aa Unfed Adult Female SG (n=5) | Aa fed Adult Female OV (n=5) | 2.4545455 | 0.11718509 | 0.23437017 |
| Aa Unfed Adult Female SG (n=5) | Aa fed Adult Female SG (n=5) | 3.1527273 | 0.07580017 | 0.17686707 |
| Aa Unfed Adult Female SG (n=5) | positve control-zymo (n=3) | 5 | 0.02534732 | 0.11828749 |
| Aa Unfed Adult Female SG (n=5) | x (n=44) | 0.2454545 | 0.62029402 | 0.75514054 |
| Aa fed Adult Female MG (n=5) | Aa fed Adult Female OV (n=5) | 0.0981818 | 0.75402253 | 0.81202426 |
| Aa fed Adult Female MG (n=5) | Aa fed Adult Female SG (n=5) | 0.0981818 | 0.75402253 | 0.81202426 |
| Aa fed Adult Female MG (n=5) | positve control-zymo (n=3) | 5 | 0.02534732 | 0.11828749 |
| Aa fed Adult Female MG (n=5) | x (n=44) | 1.7454545 | 0.18644918 | 0.30709277 |
| Aa fed Adult Female OV (n=5) | Aa fed Adult Female SG (n=5) | 0.0981818 | 0.75402253 | 0.81202426 |
| Aa fed Adult Female OV (n=5) | positve control-zymo (n=3) | 5 | 0.02534732 | 0.11828749 |
| Aa fed Adult Female OV (n=5) | x (n=44) | 1.8338182 | 0.17567723 | 0.30709277 |
| Aa fed Adult Female SG (n=5) | positve control-zymo (n=3) | 5 | 0.02534732 | 0.11828749 |
| Aa fed Adult Female SG (n=5) | x (n=44) | 1.4138182 | 0.23442359 | 0.31893534 |
| positve control-zymo (n=3) | x (n=44) | 8.25 | 0.0040752 | 0.1141056 |

Additional File 7: Kruskal-Wallis-alpha diversity (Faith_pd) pairwise among *Amblyomma americanum* tissue samples

| Group 1 | Group 2 | H | p-value | q-value |
| --- | --- | --- | --- | --- |
| Aa Unfed Adult Female MG (n=5) | Aa Unfed Adult Female OV (n=4) | 0.54 | 0.462433 | 0.542152 |
| Aa Unfed Adult Female MG (n=5) | Aa Unfed Adult Female SG (n=5) | 0.534545 | 0.464702 | 0.542152 |
| Aa Unfed Adult Female MG (n=5) | Aa fed Adult Female MG (n=5) | 5.770909 | 0.016294 | 0.189811 |
| Aa Unfed Adult Female MG (n=5) | Aa fed Adult Female OV (n=5) | 2.454545 | 0.117185 | 0.252399 |
| Aa Unfed Adult Female MG (n=5) | Aa fed Adult Female SG (n=5) | 2.454545 | 0.117185 | 0.252399 |
| Aa Unfed Adult Female MG (n=5) | positve control-zymo (n=3) | 5 | 0.025347 | 0.189811 |
| Aa Unfed Adult Female MG (n=5) | x (n=44) | 5.655273 | 0.017403 | 0.189811 |
| Aa Unfed Adult Female OV (n=4) | Aa Unfed Adult Female SG (n=5) | 0.24 | 0.624206 | 0.672222 |
| Aa Unfed Adult Female OV (n=4) | Aa fed Adult Female MG (n=5) | 4.86 | 0.027486 | 0.189811 |
| Aa Unfed Adult Female OV (n=4) | Aa fed Adult Female OV (n=5) | 2.16 | 0.141645 | 0.283289 |
| Aa Unfed Adult Female OV (n=4) | Aa fed Adult Female SG (n=5) | 1.5 | 0.220671 | 0.410241 |
| Aa Unfed Adult Female OV (n=4) | positve control-zymo (n=3) | 4.5 | 0.033895 | 0.189811 |
| Aa Unfed Adult Female OV (n=4) | x (n=44) | 3.478664 | 0.062165 | 0.235823 |
| Aa Unfed Adult Female SG (n=5) | Aa fed Adult Female MG (n=5) | 3.938182 | 0.047202 | 0.220275 |
| Aa Unfed Adult Female SG (n=5) | Aa fed Adult Female OV (n=5) | 2.454545 | 0.117185 | 0.252399 |
| Aa Unfed Adult Female SG (n=5) | Aa fed Adult Female SG (n=5) | 0.534545 | 0.464702 | 0.542152 |
| Aa Unfed Adult Female SG (n=5) | positve control-zymo (n=3) | 2.688889 | 0.10105 | 0.252399 |
| Aa Unfed Adult Female SG (n=5) | x (n=44) | 3.181091 | 0.074495 | 0.235823 |
| Aa fed Adult Female MG (n=5) | Aa fed Adult Female OV (n=5) | 0.098182 | 0.754023 | 0.781949 |
| Aa fed Adult Female MG (n=5) | Aa fed Adult Female SG (n=5) | 3.152727 | 0.0758 | 0.235823 |
| Aa fed Adult Female MG (n=5) | positve control-zymo (n=3) | 0.555556 | 0.456057 | 0.542152 |
| Aa fed Adult Female MG (n=5) | x (n=44) | 1.413818 | 0.234424 | 0.410241 |
| Aa fed Adult Female OV (n=5) | Aa fed Adult Female SG (n=5) | 1.32 | 0.250592 | 0.41274 |
| Aa fed Adult Female OV (n=5) | positve control-zymo (n=3) | 0.022222 | 0.881497 | 0.881497 |
| Aa fed Adult Female OV (n=5) | x (n=44) | 0.855273 | 0.355065 | 0.497091 |
| Aa fed Adult Female SG (n=5) | positve control-zymo (n=3) | 1.088889 | 0.296718 | 0.461561 |
| Aa fed Adult Female SG (n=5) | x (n=44) | 0.279273 | 0.597178 | 0.66884 |
| positve control-zymo (n=3) | x (n=44) | 1.001894 | 0.316853 | 0.466941 |

Additional File 8: Kruskal-Wallis-alpha diversity (Faith_pd) pairwise among *Amblyomma americanum* field-collected ticks.

| Group 1 | Group 2 | H | p-value | q-value |
| --- | --- | --- | --- | --- |
| DEPFMG (n=7) | DEPFOV (n=7) | 0.493877551 | 0.482202698 | 0.660552 |
| DEPFMG (n=7) | DEPFSG (n=7) | 0.036734694 | 0.848006214 | 0.906501 |
| DEPFMG (n=7) | DEUFMG (n=6) | 2.93877551 | 0.086476265 | 0.261628 |
| DEPFMG (n=7) | DEUFOV (n=6) | 7.367346939 | 0.006641885 | 0.16741 |
| DEPFMG (n=7) | DEUFSG (n=6) | 0.734693878 | 0.391365938 | 0.587714 |
| DEPFMG (n=7) | ILPFMG (n=5) | 4.806593407 | 0.028351037 | 0.16741 |
| DEPFMG (n=7) | ILPFOVG (n=5) | 4.120879121 | 0.042357062 | 0.192255 |
| DEPFMG (n=7) | ILPFSG (n=5) | 4.120879121 | 0.042357062 | 0.192255 |
| DEPFMG (n=7) | ILUFMG (n=5) | 3.487912088 | 0.061818506 | 0.22344 |
| DEPFMG (n=7) | ILUFOV (n=4) | 1.285714286 | 0.256839258 | 0.478582 |
| DEPFMG (n=7) | ILUFSG (n=5) | 0.797802198 | 0.37175129 | 0.569007 |
| DEPFMG (n=7) | MYPFMG (n=5) | 1.114285714 | 0.291152223 | 0.493478 |
| DEPFMG (n=7) | MYPFOV (n=3) | 0.116883117 | 0.7324399 | 0.851674 |
| DEPFMG (n=7) | MYPFSG (n=5) | 0.164835165 | 0.684743356 | 0.821692 |
| DEPFMG (n=7) | MYUFMG (n=4) | 2.285714286 | 0.130570018 | 0.331958 |
| DEPFMG (n=7) | MYUFOV (n=3) | 2.194805195 | 0.138476715 | 0.34316 |
| DEPFMG (n=7) | MYUFSG (n=4) | 0.571428571 | 0.449691798 | 0.649129 |
| DEPFMG (n=7) | Pos. control (n=4) | 7 | 0.008150972 | 0.16741 |
| DEPFOV (n=7) | DEPFSG (n=7) | 0.102040816 | 0.749394185 | 0.860102 |
| DEPFOV (n=7) | DEUFMG (n=6) | 4 | 0.045500264 | 0.192255 |
| DEPFOV (n=7) | DEUFOV (n=6) | 7.367346939 | 0.006641885 | 0.16741 |
| DEPFOV (n=7) | DEUFSG (n=6) | 2.040816327 | 0.153127451 | 0.361718 |
| DEPFOV (n=7) | ILPFMG (n=5) | 5.545054945 | 0.018532975 | 0.16741 |
| DEPFOV (n=7) | ILPFOVG (n=5) | 4.806593407 | 0.028351037 | 0.16741 |
| DEPFOV (n=7) | ILPFSG (n=5) | 4.806593407 | 0.028351037 | 0.16741 |
| DEPFOV (n=7) | ILUFMG (n=5) | 4.120879121 | 0.042357062 | 0.192255 |
| DEPFOV (n=7) | ILUFOV (n=4) | 2.285714286 | 0.130570018 | 0.331958 |
| DEPFOV (n=7) | ILUFSG (n=5) | 1.483516484 | 0.223225145 | 0.44645 |
| DEPFOV (n=7) | MYPFMG (n=5) | 2.38021978 | 0.122880258 | 0.320557 |
| DEPFOV (n=7) | MYPFOV (n=3) | 0.116883117 | 0.7324399 | 0.851674 |
| DEPFOV (n=7) | MYPFSG (n=5) | 1.483516484 | 0.223225145 | 0.44645 |
| DEPFOV (n=7) | MYUFMG (n=4) | 2.892857143 | 0.088973012 | 0.261685 |
| DEPFOV (n=7) | MYUFOV (n=3) | 2.922077922 | 0.08737528 | 0.261628 |
| DEPFOV (n=7) | MYUFSG (n=4) | 1.285714286 | 0.256839258 | 0.478582 |
| DEPFOV (n=7) | Pos. control (n=4) | 7 | 0.008150972 | 0.16741 |
| DEPFSG (n=7) | DEUFMG (n=6) | 0.510204082 | 0.475050524 | 0.656752 |
| DEPFSG (n=7) | DEUFOV (n=6) | 6.612244898 | 0.010127991 | 0.16741 |
| DEPFSG (n=7) | DEUFSG (n=6) | 0.510204082 | 0.475050524 | 0.656752 |
| DEPFSG (n=7) | ILPFMG (n=5) | 1.114285714 | 0.291152223 | 0.493478 |
| DEPFSG (n=7) | ILPFOVG (n=5) | 0.797802198 | 0.37175129 | 0.569007 |
| DEPFSG (n=7) | ILPFSG (n=5) | 0.797802198 | 0.37175129 | 0.569007 |
| DEPFSG (n=7) | ILUFMG (n=5) | 1.114285714 | 0.291152223 | 0.493478 |
| DEPFSG (n=7) | ILUFOV (n=4) | 0.571428571 | 0.449691798 | 0.649129 |
| DEPFSG (n=7) | ILUFSG (n=5) | 0.059340659 | 0.807541106 | 0.871447 |
| DEPFSG (n=7) | MYPFMG (n=5) | 0.059340659 | 0.807541106 | 0.871447 |
| DEPFSG (n=7) | MYPFOV (n=3) | 0.324675325 | 0.5688114 | 0.741234 |
| DEPFSG (n=7) | MYPFSG (n=5) | 0.059340659 | 0.807541106 | 0.871447 |
| DEPFSG (n=7) | MYUFMG (n=4) | 0.321428571 | 0.570750388 | 0.741234 |
| DEPFSG (n=7) | MYUFOV (n=3) | 0.116883117 | 0.7324399 | 0.851674 |
| DEPFSG (n=7) | MYUFSG (n=4) | 0.035714286 | 0.850106739 | 0.906501 |
| DEPFSG (n=7) | Pos. control (n=4) | 7 | 0.008150972 | 0.16741 |
| DEUFMG (n=6) | DEUFOV (n=6) | 5.025641026 | 0.024974679 | 0.16741 |
| DEUFMG (n=6) | DEUFSG (n=6) | 0.641025641 | 0.423339642 | 0.628722 |
| DEUFMG (n=6) | ILPFMG (n=5) | 1.633333333 | 0.201242621 | 0.422187 |
| DEUFMG (n=6) | ILPFOVG (n=5) | 1.2 | 0.273321678 | 0.493478 |
| DEUFMG (n=6) | ILPFSG (n=5) | 1.2 | 0.273321678 | 0.493478 |
| DEUFMG (n=6) | ILUFMG (n=5) | 0.533333333 | 0.465208818 | 0.649129 |
| DEUFMG (n=6) | ILUFOV (n=4) | 0.409090909 | 0.522431285 | 0.705988 |
| DEUFMG (n=6) | ILUFSG (n=5) | 0.133333333 | 0.715000655 | 0.847827 |
| DEUFMG (n=6) | MYPFMG (n=5) | 0.033333333 | 0.855132141 | 0.906501 |
| DEUFMG (n=6) | MYPFOV (n=3) | 2.4 | 0.12133525 | 0.319303 |
| DEUFMG (n=6) | MYPFSG (n=5) | 2.7 | 0.100348246 | 0.280695 |
| DEUFMG (n=6) | MYUFMG (n=4) | 0.045454545 | 0.83117041 | 0.893732 |
| DEUFMG (n=6) | MYUFOV (n=3) | 0.6 | 0.438578026 | 0.648145 |
| DEUFMG (n=6) | MYUFSG (n=4) | 1.136363636 | 0.286422023 | 0.493478 |
| DEUFMG (n=6) | Pos. control (n=4) | 6.545454545 | 0.010515246 | 0.16741 |
| DEUFOV (n=6) | DEUFSG (n=6) | 5.025641026 | 0.024974679 | 0.16741 |
| DEUFOV (n=6) | ILPFMG (n=5) | 3.333333333 | 0.067889155 | 0.22884 |
| DEUFOV (n=6) | ILPFOVG (n=5) | 2.7 | 0.100348246 | 0.280695 |
| DEUFOV (n=6) | ILPFSG (n=5) | 3.333333333 | 0.067889155 | 0.22884 |
| DEUFOV (n=6) | ILUFMG (n=5) | 4.8 | 0.028459737 | 0.16741 |
| DEUFOV (n=6) | ILUFOV (n=4) | 3.681818182 | 0.055008834 | 0.201252 |
| DEUFOV (n=6) | ILUFSG (n=5) | 4.033333333 | 0.044609718 | 0.192255 |
| DEUFOV (n=6) | MYPFMG (n=5) | 4.033333333 | 0.044609718 | 0.192255 |
| DEUFOV (n=6) | MYPFOV (n=3) | 4.266666667 | 0.038867104 | 0.188067 |
| DEUFOV (n=6) | MYPFSG (n=5) | 4.8 | 0.028459737 | 0.16741 |
| DEUFOV (n=6) | MYUFMG (n=4) | 2.909090909 | 0.088081512 | 0.261628 |
| DEUFOV (n=6) | MYUFOV (n=3) | 2.4 | 0.12133525 | 0.319303 |
| DEUFOV (n=6) | MYUFSG (n=4) | 4.545454545 | 0.033006258 | 0.169474 |
| DEUFOV (n=6) | Pos. control (n=4) | 5.5 | 0.019016474 | 0.16741 |
| DEUFSG (n=6) | ILPFMG (n=5) | 2.133333333 | 0.144127035 | 0.34316 |
| DEUFSG (n=6) | ILPFOVG (n=5) | 2.7 | 0.100348246 | 0.280695 |
| DEUFSG (n=6) | ILPFSG (n=5) | 2.133333333 | 0.144127035 | 0.34316 |
| DEUFSG (n=6) | ILUFMG (n=5) | 0.533333333 | 0.465208818 | 0.649129 |
| DEUFSG (n=6) | ILUFOV (n=4) | 0 | 1 | 1 |
| DEUFSG (n=6) | ILUFSG (n=5) | 0.3 | 0.583882421 | 0.75502 |
| DEUFSG (n=6) | MYPFMG (n=5) | 0.033333333 | 0.855132141 | 0.906501 |
| DEUFSG (n=6) | MYPFOV (n=3) | 1.066666667 | 0.301699582 | 0.500055 |
| DEUFSG (n=6) | MYPFSG (n=5) | 0.533333333 | 0.465208818 | 0.649129 |
| DEUFSG (n=6) | MYUFMG (n=4) | 0.727272727 | 0.393768635 | 0.587714 |
| DEUFSG (n=6) | MYUFOV (n=3) | 1.066666667 | 0.301699582 | 0.500055 |
| DEUFSG (n=6) | MYUFSG (n=4) | 0.181818182 | 0.669815358 | 0.807006 |
| DEUFSG (n=6) | Pos. control (n=4) | 6.545454545 | 0.010515246 | 0.16741 |
| ILPFMG (n=5) | ILPFOVG (n=5) | 0.272727273 | 0.601508134 | 0.770625 |
| ILPFMG (n=5) | ILPFSG (n=5) | 0.010909091 | 0.916814949 | 0.94517 |
| ILPFMG (n=5) | ILUFMG (n=5) | 0.010909091 | 0.916814949 | 0.94517 |
| ILPFMG (n=5) | ILUFOV (n=4) | 2.94 | 0.086410733 | 0.261628 |
| ILPFMG (n=5) | ILUFSG (n=5) | 3.152727273 | 0.075800175 | 0.251413 |
| ILPFMG (n=5) | MYPFMG (n=5) | 1.32 | 0.250592051 | 0.472815 |
| ILPFMG (n=5) | MYPFOV (n=3) | 5 | 0.025347319 | 0.16741 |
| ILPFMG (n=5) | MYPFSG (n=5) | 6.818181818 | 0.009023439 | 0.16741 |
| ILPFMG (n=5) | MYUFMG (n=4) | 1.5 | 0.220671362 | 0.44645 |
| ILPFMG (n=5) | MYUFOV (n=3) | 0.555555556 | 0.45605654 | 0.649129 |
| ILPFMG (n=5) | MYUFSG (n=4) | 3.84 | 0.050043521 | 0.200174 |
| ILPFMG (n=5) | Pos. control (n=4) | 6 | 0.014305878 | 0.16741 |
| ILPFOVG (n=5) | ILPFSG (n=5) | 0.010909091 | 0.916814949 | 0.94517 |
| ILPFOVG (n=5) | ILUFMG (n=5) | 0.098181818 | 0.75402253 | 0.860102 |
| ILPFOVG (n=5) | ILUFOV (n=4) | 1.5 | 0.220671362 | 0.44645 |
| ILPFOVG (n=5) | ILUFSG (n=5) | 0.883636364 | 0.347207639 | 0.548223 |
| ILPFOVG (n=5) | MYPFMG (n=5) | 1.32 | 0.250592051 | 0.472815 |
| ILPFOVG (n=5) | MYPFOV (n=3) | 3.755555556 | 0.052632303 | 0.200535 |
| ILPFOVG (n=5) | MYPFSG (n=5) | 4.810909091 | 0.028280123 | 0.16741 |
| ILPFOVG (n=5) | MYUFMG (n=4) | 0.24 | 0.624206115 | 0.770625 |
| ILPFOVG (n=5) | MYUFOV (n=3) | 0.022222222 | 0.881497452 | 0.926554 |
| ILPFOVG (n=5) | MYUFSG (n=4) | 2.16 | 0.14164469 | 0.34316 |
| ILPFOVG (n=5) | Pos. control (n=4) | 6 | 0.014305878 | 0.16741 |
| ILPFSG (n=5) | ILUFMG (n=5) | 0.098181818 | 0.75402253 | 0.860102 |
| ILPFSG (n=5) | ILUFOV (n=4) | 2.16 | 0.14164469 | 0.34316 |
| ILPFSG (n=5) | ILUFSG (n=5) | 2.454545455 | 0.117185087 | 0.316716 |
| ILPFSG (n=5) | MYPFMG (n=5) | 1.32 | 0.250592051 | 0.472815 |
| ILPFSG (n=5) | MYPFOV (n=3) | 5 | 0.025347319 | 0.16741 |
| ILPFSG (n=5) | MYPFSG (n=5) | 6.818181818 | 0.009023439 | 0.16741 |
| ILPFSG (n=5) | MYUFMG (n=4) | 0.54 | 0.462432726 | 0.649129 |
| ILPFSG (n=5) | MYUFOV (n=3) | 0.2 | 0.654720846 | 0.801699 |
| ILPFSG (n=5) | MYUFSG (n=4) | 3.84 | 0.050043521 | 0.200174 |
| ILPFSG (n=5) | Pos. control (n=4) | 6 | 0.014305878 | 0.16741 |
| ILUFMG (n=5) | ILUFOV (n=4) | 0.54 | 0.462432726 | 0.649129 |
| ILUFMG (n=5) | ILUFSG (n=5) | 0.883636364 | 0.347207639 | 0.548223 |
| ILUFMG (n=5) | MYPFMG (n=5) | 0.272727273 | 0.601508134 | 0.770625 |
| ILUFMG (n=5) | MYPFOV (n=3) | 2.688888889 | 0.101050256 | 0.280695 |
| ILUFMG (n=5) | MYPFSG (n=5) | 1.843636364 | 0.174525341 | 0.396649 |
| ILUFMG (n=5) | MYUFMG (n=4) | 0.06 | 0.806495941 | 0.871447 |
| ILUFMG (n=5) | MYUFOV (n=3) | 0.022222222 | 0.881497452 | 0.926554 |
| ILUFMG (n=5) | MYUFSG (n=4) | 1.5 | 0.220671362 | 0.44645 |
| ILUFMG (n=5) | Pos. control (n=4) | 6 | 0.014305878 | 0.16741 |
| ILUFOV (n=4) | ILUFSG (n=5) | 0.24 | 0.624206115 | 0.770625 |
| ILUFOV (n=4) | MYPFMG (n=5) | 0 | 1 | 1 |
| ILUFOV (n=4) | MYPFOV (n=3) | 1.125 | 0.288844366 | 0.493478 |
| ILUFOV (n=4) | MYPFSG (n=5) | 0.24 | 0.624206115 | 0.770625 |
| ILUFOV (n=4) | MYUFMG (n=4) | 0.083333333 | 0.772829993 | 0.865108 |
| ILUFOV (n=4) | MYUFOV (n=3) | 1.125 | 0.288844366 | 0.493478 |
| ILUFOV (n=4) | MYUFSG (n=4) | 0.333333333 | 0.563702862 | 0.741234 |
| ILUFOV (n=4) | Pos. control (n=4) | 5.333333333 | 0.020921335 | 0.16741 |
| ILUFSG (n=5) | MYPFMG (n=5) | 0.098181818 | 0.75402253 | 0.860102 |
| ILUFSG (n=5) | MYPFOV (n=3) | 2.688888889 | 0.101050256 | 0.280695 |
| ILUFSG (n=5) | MYPFSG (n=5) | 2.454545455 | 0.117185087 | 0.316716 |
| ILUFSG (n=5) | MYUFMG (n=4) | 0.24 | 0.624206115 | 0.770625 |
| ILUFSG (n=5) | MYUFOV (n=3) | 0.555555556 | 0.45605654 | 0.649129 |
| ILUFSG (n=5) | MYUFSG (n=4) | 0.24 | 0.624206115 | 0.770625 |
| ILUFSG (n=5) | Pos. control (n=4) | 6 | 0.014305878 | 0.16741 |
| MYPFMG (n=5) | MYPFOV (n=3) | 1.8 | 0.179712495 | 0.399361 |
| MYPFMG (n=5) | MYPFSG (n=5) | 1.843636364 | 0.174525341 | 0.396649 |
| MYPFMG (n=5) | MYUFMG (n=4) | 0.06 | 0.806495941 | 0.871447 |
| MYPFMG (n=5) | MYUFOV (n=3) | 1.088888889 | 0.296717526 | 0.500055 |
| MYPFMG (n=5) | MYUFSG (n=4) | 0.96 | 0.327186878 | 0.527721 |
| MYPFMG (n=5) | Pos. control (n=4) | 6 | 0.014305878 | 0.16741 |
| MYPFOV (n=3) | MYPFSG (n=5) | 0.555555556 | 0.45605654 | 0.649129 |
| MYPFOV (n=3) | MYUFMG (n=4) | 3.125 | 0.077099872 | 0.251413 |
| MYPFOV (n=3) | MYUFOV (n=3) | 3.857142857 | 0.049534613 | 0.200174 |
| MYPFOV (n=3) | MYUFSG (n=4) | 1.125 | 0.288844366 | 0.493478 |
| MYPFOV (n=3) | Pos. control (n=4) | 4.5 | 0.033894854 | 0.169474 |
| MYPFSG (n=5) | MYUFMG (n=4) | 1.5 | 0.220671362 | 0.44645 |
| MYPFSG (n=5) | MYUFOV (n=3) | 5 | 0.025347319 | 0.16741 |
| MYPFSG (n=5) | MYUFSG (n=4) | 0.06 | 0.806495941 | 0.871447 |
| MYPFSG (n=5) | Pos. control (n=4) | 6 | 0.014305878 | 0.16741 |
| MYUFMG (n=4) | MYUFOV (n=3) | 0.125 | 0.72367361 | 0.851381 |
| MYUFMG (n=4) | MYUFSG (n=4) | 1.333333333 | 0.248213079 | 0.472815 |
| MYUFMG (n=4) | Pos. control (n=4) | 5.333333333 | 0.020921335 | 0.16741 |
| MYUFOV (n=3) | MYUFSG (n=4) | 2 | 0.157299207 | 0.362998 |
| MYUFOV (n=3) | Pos. control (n=4) | 4.5 | 0.033894854 | 0.169474 |
| MYUFSG (n=4) | Pos. control (n=4) | 5.333333333 | 0.020921335 | 0.16741 |

Additional File 9: Kruskal-Wallis-alpha diversity (evenness) pairwise among *Amblyomma americanum* field-collected ticks.

| Group 1 | Group 2 | H | p-value | q-value |
| --- | --- | --- | --- | --- |
| DEPFMG (n=7) | DEPFOV (n=7) | 0.036734694 | 0.848006214 | 0.904368871 |
| DEPFMG (n=7) | DEPFSG (n=7) | 0.689795918 | 0.406233805 | 0.569486643 |
| DEPFMG (n=7) | DEUFMG (n=6) | 5.224489796 | 0.022270979 | 0.243940602 |
| DEPFMG (n=7) | DEUFOV (n=6) | 6.612244898 | 0.010127991 | 0.210304919 |
| DEPFMG (n=7) | DEUFSG (n=6) | 2.040816327 | 0.153127451 | 0.386035591 |
| DEPFMG (n=7) | ILPFMG (n=5) | 1.483516484 | 0.223225145 | 0.413379899 |
| DEPFMG (n=7) | ILPFOVG (n=5) | 2.38021978 | 0.122880258 | 0.344524088 |
| DEPFMG (n=7) | ILPFSG (n=5) | 2.38021978 | 0.122880258 | 0.344524088 |
| DEPFMG (n=7) | ILUFMG (n=5) | 1.114285714 | 0.291152223 | 0.450235397 |
| DEPFMG (n=7) | ILUFOV (n=4) | 1.285714286 | 0.256839258 | 0.442826307 |
| DEPFMG (n=7) | ILUFSG (n=5) | 0.323076923 | 0.569764368 | 0.71819038 |
| DEPFMG (n=7) | MYPFMG (n=5) | 1.114285714 | 0.291152223 | 0.450235397 |
| DEPFMG (n=7) | MYPFOV (n=3) | 0.012987013 | 0.909269045 | 0.96049547 |
| DEPFMG (n=7) | MYPFSG (n=5) | 0.006593407 | 0.935283063 | 0.974253191 |
| DEPFMG (n=7) | MYUFMG (n=4) | 1.285714286 | 0.256839258 | 0.442826307 |
| DEPFMG (n=7) | MYUFOV (n=3) | 0.116883117 | 0.7324399 | 0.832318068 |
| DEPFMG (n=7) | MYUFSG (n=4) | 0.035714286 | 0.850106739 | 0.904368871 |
| DEPFMG (n=7) | Pos. control (n=4) | 0.571428571 | 0.449691798 | 0.612116866 |
| DEPFOV (n=7) | DEPFSG (n=7) | 0.689795918 | 0.406233805 | 0.569486643 |
| DEPFOV (n=7) | DEUFMG (n=6) | 9 | 0.002699796 | 0.210304919 |
| DEPFOV (n=7) | DEUFOV (n=6) | 9 | 0.002699796 | 0.210304919 |
| DEPFOV (n=7) | DEUFSG (n=6) | 2.93877551 | 0.086476265 | 0.299909028 |
| DEPFOV (n=7) | ILPFMG (n=5) | 2.907692308 | 0.088157935 | 0.299909028 |
| DEPFOV (n=7) | ILPFOVG (n=5) | 6.336263736 | 0.01182938 | 0.221800877 |
| DEPFOV (n=7) | ILPFSG (n=5) | 3.487912088 | 0.061818506 | 0.282871479 |
| DEPFOV (n=7) | ILUFMG (n=5) | 1.905494505 | 0.167464596 | 0.396424621 |
| DEPFOV (n=7) | ILUFOV (n=4) | 2.285714286 | 0.130570018 | 0.359367022 |
| DEPFOV (n=7) | ILUFSG (n=5) | 1.905494505 | 0.167464596 | 0.396424621 |
| DEPFOV (n=7) | MYPFMG (n=5) | 2.907692308 | 0.088157935 | 0.299909028 |
| DEPFOV (n=7) | MYPFOV (n=3) | 1.051948052 | 0.305058859 | 0.464556638 |
| DEPFOV (n=7) | MYPFSG (n=5) | 0.006593407 | 0.935283063 | 0.974253191 |
| DEPFOV (n=7) | MYUFMG (n=4) | 2.285714286 | 0.130570018 | 0.359367022 |
| DEPFOV (n=7) | MYUFOV (n=3) | 0.116883117 | 0.7324399 | 0.832318068 |
| DEPFOV (n=7) | MYUFSG (n=4) | 0.035714286 | 0.850106739 | 0.904368871 |
| DEPFOV (n=7) | Pos. control (n=4) | 1.75 | 0.185876732 | 0.402485242 |
| DEPFSG (n=7) | DEUFMG (n=6) | 5.224489796 | 0.022270979 | 0.243940602 |
| DEPFSG (n=7) | DEUFOV (n=6) | 6.612244898 | 0.010127991 | 0.210304919 |
| DEPFSG (n=7) | DEUFSG (n=6) | 1.653061224 | 0.198542794 | 0.402485242 |
| DEPFSG (n=7) | ILPFMG (n=5) | 1.483516484 | 0.223225145 | 0.413379899 |
| DEPFSG (n=7) | ILPFOVG (n=5) | 1.905494505 | 0.167464596 | 0.396424621 |
| DEPFSG (n=7) | ILPFSG (n=5) | 1.905494505 | 0.167464596 | 0.396424621 |
| DEPFSG (n=7) | ILUFMG (n=5) | 0.059340659 | 0.807541106 | 0.877762072 |
| DEPFSG (n=7) | ILUFOV (n=4) | 0.892857143 | 0.344704222 | 0.506917974 |
| DEPFSG (n=7) | ILUFSG (n=5) | 0.006593407 | 0.935283063 | 0.974253191 |
| DEPFSG (n=7) | MYPFMG (n=5) | 0.534065934 | 0.464902455 | 0.612116866 |
| DEPFSG (n=7) | MYPFOV (n=3) | 1.051948052 | 0.305058859 | 0.464556638 |
| DEPFSG (n=7) | MYPFSG (n=5) | 1.114285714 | 0.291152223 | 0.450235397 |
| DEPFSG (n=7) | MYUFMG (n=4) | 0.892857143 | 0.344704222 | 0.506917974 |
| DEPFSG (n=7) | MYUFOV (n=3) | 0.116883117 | 0.7324399 | 0.832318068 |
| DEPFSG (n=7) | MYUFSG (n=4) | 1.285714286 | 0.256839258 | 0.442826307 |
| DEPFSG (n=7) | Pos. control (n=4) | 2.892857143 | 0.088973012 | 0.299909028 |
| DEUFMG (n=6) | DEUFOV (n=6) | 0.923076923 | 0.336668368 | 0.505002551 |
| DEUFMG (n=6) | DEUFSG (n=6) | 2.564102564 | 0.109314576 | 0.331256292 |
| DEUFMG (n=6) | ILPFMG (n=5) | 3.333333333 | 0.067889155 | 0.282871479 |
| DEUFMG (n=6) | ILPFOVG (n=5) | 1.2 | 0.273321678 | 0.450235397 |
| DEUFMG (n=6) | ILPFSG (n=5) | 1.2 | 0.273321678 | 0.450235397 |
| DEUFMG (n=6) | ILUFMG (n=5) | 3.333333333 | 0.067889155 | 0.282871479 |
| DEUFMG (n=6) | ILUFOV (n=4) | 1.636363636 | 0.200825123 | 0.402485242 |
| DEUFMG (n=6) | ILUFSG (n=5) | 7.5 | 0.006169899 | 0.210304919 |
| DEUFMG (n=6) | MYPFMG (n=5) | 2.7 | 0.100348246 | 0.312526565 |
| DEUFMG (n=6) | MYPFOV (n=3) | 5.4 | 0.020136752 | 0.243940602 |
| DEUFMG (n=6) | MYPFSG (n=5) | 5.633333333 | 0.017622091 | 0.243940602 |
| DEUFMG (n=6) | MYUFMG (n=4) | 0.727272727 | 0.393768635 | 0.557219766 |
| DEUFMG (n=6) | MYUFOV (n=3) | 5.4 | 0.020136752 | 0.243940602 |
| DEUFMG (n=6) | MYUFSG (n=4) | 6.545454545 | 0.010515246 | 0.210304919 |
| DEUFMG (n=6) | Pos. control (n=4) | 6.545454545 | 0.010515246 | 0.210304919 |
| DEUFOV (n=6) | DEUFSG (n=6) | 3.692307692 | 0.054663936 | 0.273319679 |
| DEUFOV (n=6) | ILPFMG (n=5) | 2.7 | 0.100348246 | 0.312526565 |
| DEUFOV (n=6) | ILPFOVG (n=5) | 3.333333333 | 0.067889155 | 0.282871479 |
| DEUFOV (n=6) | ILPFSG (n=5) | 2.7 | 0.100348246 | 0.312526565 |
| DEUFOV (n=6) | ILUFMG (n=5) | 4.8 | 0.028459737 | 0.243940602 |
| DEUFOV (n=6) | ILUFOV (n=4) | 1.636363636 | 0.200825123 | 0.402485242 |
| DEUFOV (n=6) | ILUFSG (n=5) | 7.5 | 0.006169899 | 0.210304919 |
| DEUFOV (n=6) | MYPFMG (n=5) | 4.033333333 | 0.044609718 | 0.26851277 |
| DEUFOV (n=6) | MYPFOV (n=3) | 5.4 | 0.020136752 | 0.243940602 |
| DEUFOV (n=6) | MYPFSG (n=5) | 4.8 | 0.028459737 | 0.243940602 |
| DEUFOV (n=6) | MYUFMG (n=4) | 1.636363636 | 0.200825123 | 0.402485242 |
| DEUFOV (n=6) | MYUFOV (n=3) | 5.4 | 0.020136752 | 0.243940602 |
| DEUFOV (n=6) | MYUFSG (n=4) | 6.545454545 | 0.010515246 | 0.210304919 |
| DEUFOV (n=6) | Pos. control (n=4) | 6.545454545 | 0.010515246 | 0.210304919 |
| DEUFSG (n=6) | ILPFMG (n=5) | 0.133333333 | 0.715000655 | 0.83139611 |
| DEUFSG (n=6) | ILPFOVG (n=5) | 0.133333333 | 0.715000655 | 0.83139611 |
| DEUFSG (n=6) | ILPFSG (n=5) | 0.3 | 0.583882421 | 0.732906804 |
| DEUFSG (n=6) | ILUFMG (n=5) | 0.533333333 | 0.465208818 | 0.612116866 |
| DEUFSG (n=6) | ILUFOV (n=4) | 0 | 1 | 1 |
| DEUFSG (n=6) | ILUFSG (n=5) | 1.2 | 0.273321678 | 0.450235397 |
| DEUFSG (n=6) | MYPFMG (n=5) | 0.033333333 | 0.855132141 | 0.906500502 |
| DEUFSG (n=6) | MYPFOV (n=3) | 2.4 | 0.12133525 | 0.344524088 |
| DEUFSG (n=6) | MYPFSG (n=5) | 2.7 | 0.100348246 | 0.312526565 |
| DEUFSG (n=6) | MYUFMG (n=4) | 0 | 1 | 1 |
| DEUFSG (n=6) | MYUFOV (n=3) | 2.4 | 0.12133525 | 0.344524088 |
| DEUFSG (n=6) | MYUFSG (n=4) | 2.227272727 | 0.135593001 | 0.366467571 |
| DEUFSG (n=6) | Pos. control (n=4) | 4.545454545 | 0.033006258 | 0.26851277 |
| ILPFMG (n=5) | ILPFOVG (n=5) | 0.098181818 | 0.75402253 | 0.847216326 |
| ILPFMG (n=5) | ILPFSG (n=5) | 0.534545455 | 0.4647021 | 0.612116866 |
| ILPFMG (n=5) | ILUFMG (n=5) | 0.272727273 | 0.601508134 | 0.748765313 |
| ILPFMG (n=5) | ILUFOV (n=4) | 0.54 | 0.462432726 | 0.612116866 |
| ILPFMG (n=5) | ILUFSG (n=5) | 1.32 | 0.250592051 | 0.442221266 |
| ILPFMG (n=5) | MYPFMG (n=5) | 0.098181818 | 0.75402253 | 0.847216326 |
| ILPFMG (n=5) | MYPFOV (n=3) | 1.8 | 0.179712495 | 0.396424621 |
| ILPFMG (n=5) | MYPFSG (n=5) | 2.454545455 | 0.117185087 | 0.344524088 |
| ILPFMG (n=5) | MYUFMG (n=4) | 0.24 | 0.624206115 | 0.761226969 |
| ILPFMG (n=5) | MYUFOV (n=3) | 1.8 | 0.179712495 | 0.396424621 |
| ILPFMG (n=5) | MYUFSG (n=4) | 2.94 | 0.086410733 | 0.299909028 |
| ILPFMG (n=5) | Pos. control (n=4) | 3.84 | 0.050043521 | 0.26851277 |
| ILPFOVG (n=5) | ILPFSG (n=5) | 0.010909091 | 0.916814949 | 0.965068367 |
| ILPFOVG (n=5) | ILUFMG (n=5) | 1.32 | 0.250592051 | 0.442221266 |
| ILPFOVG (n=5) | ILUFOV (n=4) | 0 | 1 | 1 |
| ILPFOVG (n=5) | ILUFSG (n=5) | 3.152727273 | 0.075800175 | 0.299909028 |
| ILPFOVG (n=5) | MYPFMG (n=5) | 0.534545455 | 0.4647021 | 0.612116866 |
| ILPFOVG (n=5) | MYPFOV (n=3) | 5 | 0.025347319 | 0.243940602 |
| ILPFOVG (n=5) | MYPFSG (n=5) | 2.454545455 | 0.117185087 | 0.344524088 |
| ILPFOVG (n=5) | MYUFMG (n=4) | 0 | 1 | 1 |
| ILPFOVG (n=5) | MYUFOV (n=3) | 5 | 0.025347319 | 0.243940602 |
| ILPFOVG (n=5) | MYUFSG (n=4) | 4.86 | 0.027486336 | 0.243940602 |
| ILPFOVG (n=5) | Pos. control (n=4) | 6 | 0.014305878 | 0.243940602 |
| ILPFSG (n=5) | ILUFMG (n=5) | 0.534545455 | 0.4647021 | 0.612116866 |
| ILPFSG (n=5) | ILUFOV (n=4) | 0.06 | 0.806495941 | 0.877762072 |
| ILPFSG (n=5) | ILUFSG (n=5) | 2.454545455 | 0.117185087 | 0.344524088 |
| ILPFSG (n=5) | MYPFMG (n=5) | 0.534545455 | 0.4647021 | 0.612116866 |
| ILPFSG (n=5) | MYPFOV (n=3) | 1.8 | 0.179712495 | 0.396424621 |
| ILPFSG (n=5) | MYPFSG (n=5) | 3.152727273 | 0.075800175 | 0.299909028 |
| ILPFSG (n=5) | MYUFMG (n=4) | 0 | 1 | 1 |
| ILPFSG (n=5) | MYUFOV (n=3) | 1.8 | 0.179712495 | 0.396424621 |
| ILPFSG (n=5) | MYUFSG (n=4) | 2.94 | 0.086410733 | 0.299909028 |
| ILPFSG (n=5) | Pos. control (n=4) | 3.84 | 0.050043521 | 0.26851277 |
| ILUFMG (n=5) | ILUFOV (n=4) | 0.24 | 0.624206115 | 0.761226969 |
| ILUFMG (n=5) | ILUFSG (n=5) | 0.272727273 | 0.601508134 | 0.748765313 |
| ILUFMG (n=5) | MYPFMG (n=5) | 0.098181818 | 0.75402253 | 0.847216326 |
| ILUFMG (n=5) | MYPFOV (n=3) | 2.688888889 | 0.101050256 | 0.312526565 |
| ILUFMG (n=5) | MYPFSG (n=5) | 1.843636364 | 0.174525341 | 0.396424621 |
| ILUFMG (n=5) | MYUFMG (n=4) | 0.54 | 0.462432726 | 0.612116866 |
| ILUFMG (n=5) | MYUFOV (n=3) | 1.088888889 | 0.296717526 | 0.456488502 |
| ILUFMG (n=5) | MYUFSG (n=4) | 1.5 | 0.220671362 | 0.413379899 |
| ILUFMG (n=5) | Pos. control (n=4) | 4.86 | 0.027486336 | 0.243940602 |
| ILUFOV (n=4) | ILUFSG (n=5) | 1.5 | 0.220671362 | 0.413379899 |
| ILUFOV (n=4) | MYPFMG (n=5) | 0.06 | 0.806495941 | 0.877762072 |
| ILUFOV (n=4) | MYPFOV (n=3) | 1.125 | 0.288844366 | 0.450235397 |
| ILUFOV (n=4) | MYPFSG (n=5) | 2.16 | 0.14164469 | 0.372749185 |
| ILUFOV (n=4) | MYUFMG (n=4) | 0.083333333 | 0.772829993 | 0.861892185 |
| ILUFOV (n=4) | MYUFOV (n=3) | 1.125 | 0.288844366 | 0.450235397 |
| ILUFOV (n=4) | MYUFSG (n=4) | 2.083333333 | 0.148914673 | 0.381832495 |
| ILUFOV (n=4) | Pos. control (n=4) | 3 | 0.083264517 | 0.299909028 |
| ILUFSG (n=5) | MYPFMG (n=5) | 1.32 | 0.250592051 | 0.442221266 |
| ILUFSG (n=5) | MYPFOV (n=3) | 1.8 | 0.179712495 | 0.396424621 |
| ILUFSG (n=5) | MYPFSG (n=5) | 0.883636364 | 0.347207639 | 0.508108741 |
| ILUFSG (n=5) | MYUFMG (n=4) | 1.5 | 0.220671362 | 0.413379899 |
| ILUFSG (n=5) | MYUFOV (n=3) | 1.8 | 0.179712495 | 0.396424621 |
| ILUFSG (n=5) | MYUFSG (n=4) | 1.5 | 0.220671362 | 0.413379899 |
| ILUFSG (n=5) | Pos. control (n=4) | 3.84 | 0.050043521 | 0.26851277 |
| MYPFMG (n=5) | MYPFOV (n=3) | 1.8 | 0.179712495 | 0.396424621 |
| MYPFMG (n=5) | MYPFSG (n=5) | 1.32 | 0.250592051 | 0.442221266 |
| MYPFMG (n=5) | MYUFMG (n=4) | 0.24 | 0.624206115 | 0.761226969 |
| MYPFMG (n=5) | MYUFOV (n=3) | 1.8 | 0.179712495 | 0.396424621 |
| MYPFMG (n=5) | MYUFSG (n=4) | 2.16 | 0.14164469 | 0.372749185 |
| MYPFMG (n=5) | Pos. control (n=4) | 3.84 | 0.050043521 | 0.26851277 |
| MYPFOV (n=3) | MYPFSG (n=5) | 0.2 | 0.654720846 | 0.788820296 |
| MYPFOV (n=3) | MYUFMG (n=4) | 1.125 | 0.288844366 | 0.450235397 |
| MYPFOV (n=3) | MYUFOV (n=3) | 3.857142857 | 0.049534613 | 0.26851277 |
| MYPFOV (n=3) | MYUFSG (n=4) | 0 | 1 | 1 |
| MYPFOV (n=3) | Pos. control (n=4) | 0.5 | 0.479500122 | 0.625434942 |
| MYPFSG (n=5) | MYUFMG (n=4) | 2.16 | 0.14164469 | 0.372749185 |
| MYPFSG (n=5) | MYUFOV (n=3) | 0.2 | 0.654720846 | 0.788820296 |
| MYPFSG (n=5) | MYUFSG (n=4) | 0.06 | 0.806495941 | 0.877762072 |
| MYPFSG (n=5) | Pos. control (n=4) | 0.96 | 0.327186878 | 0.493246549 |
| MYUFMG (n=4) | MYUFOV (n=3) | 1.125 | 0.288844366 | 0.450235397 |
| MYUFMG (n=4) | MYUFSG (n=4) | 2.083333333 | 0.148914673 | 0.381832495 |
| MYUFMG (n=4) | Pos. control (n=4) | 3 | 0.083264517 | 0.299909028 |
| MYUFOV (n=3) | MYUFSG (n=4) | 0 | 1 | 1 |
| MYUFOV (n=3) | Pos. control (n=4) | 3.125 | 0.077099872 | 0.299909028 |
| MYUFSG (n=4) | Pos. control (n=4) | 1.333333333 | 0.248213079 | 0.442221266 |

Additional File 10: Bray-Curtis beta diversity distances of Amblyomma americanum Life stages from lab-raised ticks (pairwise)

| Group 1 | Group 2 | pseudo-F | p-value | q-value |
| --- | --- | --- | --- | --- |
| Aa-Egg | Aa-F-Female | 0.900403 | 0.577 | 0.610288 |
| Aa-Egg | Aa-F-Male | 2.271165 | 0.056 | 0.146667 |
| Aa-Egg | Aa-F-Nymph | 0.874892 | 0.52 | 0.572 |
| Aa-Egg | Aa-F-larvae | 1.343534 | 0.18 | 0.303333 |
| Aa-Egg | Aa-U-Female | 1.368716 | 0.182 | 0.303333 |
| Aa-Egg | Aa-U-Male | 0.9192 | 0.489 | 0.560313 |
| Aa-Egg | Aa-UF-Nymph | 1.680759 | 0.089 | 0.194615 |
| Aa-Egg | Aa-UF-larvae | 1.244726 | 0.215 | 0.337857 |
| Aa-Egg | Positive control | 298.7562 | 0.019 | 0.101538 |
| Aa-Egg | x | 0.552784 | 0.327 | 0.432143 |
| Aa-F-Female | Aa-F-Male | 2.114509 | 0.012 | 0.101538 |
| Aa-F-Female | Aa-F-Nymph | 1.022734 | 0.408 | 0.492609 |
| Aa-F-Female | Aa-F-larvae | 0.776815 | 0.776 | 0.79037 |
| Aa-F-Female | Aa-U-Female | 1.367496 | 0.16 | 0.283871 |
| Aa-F-Female | Aa-U-Male | 1.302243 | 0.19 | 0.307353 |
| Aa-F-Female | Aa-UF-Nymph | 1.651148 | 0.041 | 0.1375 |
| Aa-F-Female | Aa-UF-larvae | 1.482442 | 0.149 | 0.273167 |
| Aa-F-Female | Positive control | 185.1981 | 0.022 | 0.101538 |
| Aa-F-Female | x | 0.599164 | 0.32 | 0.432143 |
| Aa-F-Male | Aa-F-Nymph | 1.97959 | 0.034 | 0.133571 |
| Aa-F-Male | Aa-F-larvae | 3.347831 | 0.005 | 0.101538 |
| Aa-F-Male | Aa-U-Female | 1.679402 | 0.049 | 0.141842 |
| Aa-F-Male | Aa-U-Male | 1.859615 | 0.068 | 0.155833 |
| Aa-F-Male | Aa-UF-Nymph | 1.144691 | 0.261 | 0.387973 |
| Aa-F-Male | Aa-UF-larvae | 1.144877 | 0.33 | 0.432143 |
| Aa-F-Male | Positive control | 457.6598 | 0.019 | 0.101538 |
| Aa-F-Male | x | 0.504533 | 0.412 | 0.492609 |
| Aa-F-Nymph | Aa-F-larvae | 1.658671 | 0.105 | 0.213889 |
| Aa-F-Nymph | Aa-U-Female | 2.097648 | 0.045 | 0.1375 |
| Aa-F-Nymph | Aa-U-Male | 1.915473 | 0.065 | 0.155435 |
| Aa-F-Nymph | Aa-UF-Nymph | 1.559447 | 0.092 | 0.194615 |
| Aa-F-Nymph | Aa-UF-larvae | 0.930011 | 0.503 | 0.564592 |
| Aa-F-Nymph | Positive control | 279.0062 | 0.019 | 0.101538 |
| Aa-F-Nymph | x | 0.524473 | 0.408 | 0.492609 |
| Aa-F-larvae | Aa-U-Female | 2.328956 | 0.06 | 0.15 |
| Aa-F-larvae | Aa-U-Male | 1.516431 | 0.144 | 0.273167 |
| Aa-F-larvae | Aa-UF-Nymph | 2.633105 | 0.014 | 0.101538 |
| Aa-F-larvae | Aa-UF-larvae | 2.441847 | 0.056 | 0.146667 |
| Aa-F-larvae | Positive control | 177.9186 | 0.024 | 0.101538 |
| Aa-F-larvae | x | 0.674975 | 0.302 | 0.432143 |
| Aa-U-Female | Aa-U-Male | 0.315868 | 0.969 | 0.969 |
| Aa-U-Female | Aa-UF-Nymph | 1.461671 | 0.147 | 0.273167 |
| Aa-U-Female | Aa-UF-larvae | 2.047734 | 0.045 | 0.1375 |
| Aa-U-Female | Positive control | 325.1499 | 0.016 | 0.101538 |
| Aa-U-Female | x | 0.550333 | 0.327 | 0.432143 |
| Aa-U-Male | Aa-UF-Nymph | 1.241171 | 0.26 | 0.387973 |
| Aa-U-Male | Aa-UF-larvae | 1.95976 | 0.045 | 0.1375 |
| Aa-U-Male | Positive control | 302.5602 | 0.024 | 0.101538 |
| Aa-U-Male | x | 0.392005 | 0.531 | 0.572647 |
| Aa-UF-Nymph | Aa-UF-larvae | 0.91418 | 0.455 | 0.532447 |
| Aa-UF-Nymph | Positive control | 375.0475 | 0.018 | 0.101538 |
| Aa-UF-Nymph | x | 0.470076 | 0.603 | 0.625755 |
| Aa-UF-larvae | Positive control | 349.5493 | 0.019 | 0.101538 |
| Aa-UF-larvae | x | 0.531474 | 0.356 | 0.455349 |
| Positive control | x | 33.7836 | 0.001 | 0.055 |

Additional File 11: Bray-Curtis beta diversity distances of Amblyomma americanum tissues (pairwise)

| **Group 1** | **Group 2** | **pseudo-F** | **p-value** | **q-value** |
| --- | --- | --- | --- | --- |
| Aa Unfed Adult Female MG | Aa Unfed Adult Female OV | 6.4825176 | 0.086 | 0.1465882 |
| Aa Unfed Adult Female MG | Aa Unfed Adult Female SG | 1.1256179 | 0.414 | 0.462 |
| Aa Unfed Adult Female MG | Aa fed Adult Female MG | 2.8586093 | 0.009 | 0.063 |
| Aa Unfed Adult Female MG | Aa fed Adult Female OV | 1.3742511 | 0.162 | 0.216 |
| Aa Unfed Adult Female MG | Aa fed Adult Female SG | 1.8287407 | 0.023 | 0.0715556 |
| Aa Unfed Adult Female MG | positve control-zymo | 222.10439 | 0.022 | 0.0715556 |
| Aa Unfed Adult Female MG | x | 1.7134831 | 0.087 | 0.1465882 |
| Aa Unfed Adult Female OV | Aa Unfed Adult Female SG | 3.7422075 | 0.336 | 0.392 |
| Aa Unfed Adult Female OV | Aa fed Adult Female MG | 7.3136473 | 0.036 | 0.084 |
| Aa Unfed Adult Female OV | Aa fed Adult Female OV | 5.5196671 | 0.086 | 0.1465882 |
| Aa Unfed Adult Female OV | Aa fed Adult Female SG | 7.2348568 | 0.034 | 0.084 |
| Aa Unfed Adult Female OV | positve control-zymo | 6.9235051 | 0.089 | 0.1465882 |
| Aa Unfed Adult Female OV | x | 55.129258 | 0.002 | 0.028 |
| Aa Unfed Adult Female SG | Aa fed Adult Female MG | 2.2086595 | 0.062 | 0.1335385 |
| Aa Unfed Adult Female SG | Aa fed Adult Female OV | 1.2871189 | 0.255 | 0.3245455 |
| Aa Unfed Adult Female SG | Aa fed Adult Female SG | 0.976306 | 0.429 | 0.462 |
| Aa Unfed Adult Female SG | positve control-zymo | 239.50425 | 0.105 | 0.1547368 |
| Aa Unfed Adult Female SG | x | 1.0826691 | 0.282 | 0.3433043 |
| Aa fed Adult Female MG | Aa fed Adult Female OV | 1.5860317 | 0.02 | 0.0715556 |
| Aa fed Adult Female MG | Aa fed Adult Female SG | 2.7915847 | 0.007 | 0.063 |
| Aa fed Adult Female MG | positve control-zymo | 463.67094 | 0.017 | 0.0715556 |
| Aa fed Adult Female MG | x | 1.6372301 | 0.101 | 0.1547368 |
| Aa fed Adult Female OV | Aa fed Adult Female SG | 1.4688 | 0.12 | 0.168 |
| Aa fed Adult Female OV | positve control-zymo | 369.10453 | 0.036 | 0.084 |
| Aa fed Adult Female OV | x | 0.7471964 | 0.626 | 0.626 |
| Aa fed Adult Female SG | positve control-zymo | 380.92309 | 0.016 | 0.0715556 |
| Aa fed Adult Female SG | x | 0.8952159 | 0.491 | 0.5091852 |
| positve control-zymo | x | 379.83327 | 0.001 | 0.028 |

Additional File 12: Weighted UniFrac diversity distances of *Amblyomma americanum* field-collected ticks (pairwise).

| Group 1 | Group 2 | Sample size | Permutations | pseudo-F | p-value | q-value |
| --- | --- | --- | --- | --- | --- | --- |
| DEPFMG | DEPFOV | 14 | 999 | 0.938172 | 0.624 | 0.690775 |
| DEPFMG | DEPFSG | 14 | 999 | 0.78683 | 0.812 | 0.842907 |
| DEPFMG | DEUFMG | 13 | 999 | 1.29769 | 0.084 | 0.161538 |
| DEPFMG | DEUFOV | 13 | 999 | 2.920394 | 0.002 | 0.066667 |
| DEPFMG | DEUFSG | 13 | 999 | 0.982818 | 0.442 | 0.52619 |
| DEPFMG | ILPFMG | 12 | 999 | 1.646114 | 0.018 | 0.078261 |
| DEPFMG | ILPFOVG | 12 | 999 | 1.534118 | 0.029 | 0.093548 |
| DEPFMG | ILPFSG | 12 | 999 | 1.44896 | 0.061 | 0.133577 |
| DEPFMG | ILUFMG | 12 | 999 | 1.366354 | 0.055 | 0.126923 |
| DEPFMG | ILUFOV | 11 | 999 | 1.059968 | 0.306 | 0.39913 |
| DEPFMG | ILUFSG | 12 | 999 | 1.135637 | 0.213 | 0.302844 |
| DEPFMG | MYPFMG | 12 | 999 | 1.099226 | 0.263 | 0.360274 |
| DEPFMG | MYPFOV | 10 | 999 | 1.048404 | 0.317 | 0.409914 |
| DEPFMG | MYPFSG | 12 | 999 | 0.862803 | 0.774 | 0.809059 |
| DEPFMG | MYUFMG | 11 | 999 | 1.471875 | 0.047 | 0.116529 |
| DEPFMG | MYUFOV | 10 | 999 | 1.126066 | 0.21 | 0.3 |
| DEPFMG | MYUFSG | 11 | 999 | 1.018175 | 0.437 | 0.522311 |
| DEPFMG | Pos. control | 11 | 999 | 6.921602 | 0.005 | 0.066667 |
| DEPFOV | DEPFSG | 14 | 999 | 0.839008 | 0.695 | 0.735211 |
| DEPFOV | DEUFMG | 13 | 999 | 1.614655 | 0.014 | 0.072414 |
| DEPFOV | DEUFOV | 13 | 999 | 3.124407 | 0.002 | 0.066667 |
| DEPFOV | DEUFSG | 13 | 999 | 1.197787 | 0.13 | 0.217877 |
| DEPFOV | ILPFMG | 12 | 999 | 1.93078 | 0.021 | 0.080769 |
| DEPFOV | ILPFOVG | 12 | 999 | 1.876178 | 0.008 | 0.066667 |
| DEPFOV | ILPFSG | 12 | 999 | 2.078866 | 0.008 | 0.066667 |
| DEPFOV | ILUFMG | 12 | 999 | 1.533421 | 0.04 | 0.105263 |
| DEPFOV | ILUFOV | 11 | 999 | 1.178785 | 0.166 | 0.255385 |
| DEPFOV | ILUFSG | 12 | 999 | 1.080093 | 0.25 | 0.345622 |
| DEPFOV | MYPFMG | 12 | 999 | 1.206547 | 0.183 | 0.2745 |
| DEPFOV | MYPFOV | 10 | 999 | 0.855338 | 0.773 | 0.809059 |
| DEPFOV | MYPFSG | 12 | 999 | 0.953455 | 0.543 | 0.625287 |
| DEPFOV | MYUFMG | 11 | 999 | 1.470334 | 0.05 | 0.120968 |
| DEPFOV | MYUFOV | 10 | 999 | 1.228336 | 0.146 | 0.238043 |
| DEPFOV | MYUFSG | 11 | 999 | 0.897805 | 0.636 | 0.696 |
| DEPFOV | Pos. control | 11 | 999 | 6.886684 | 0.004 | 0.066667 |
| DEPFSG | DEUFMG | 13 | 999 | 1.233024 | 0.153 | 0.241579 |
| DEPFSG | DEUFOV | 13 | 999 | 2.164504 | 0.012 | 0.072414 |
| DEPFSG | DEUFSG | 13 | 999 | 0.871116 | 0.66 | 0.714801 |
| DEPFSG | ILPFMG | 12 | 999 | 1.347355 | 0.13 | 0.217877 |
| DEPFSG | ILPFOVG | 12 | 999 | 1.160979 | 0.206 | 0.295694 |
| DEPFSG | ILPFSG | 12 | 999 | 1.240233 | 0.166 | 0.255385 |
| DEPFSG | ILUFMG | 12 | 999 | 1.074741 | 0.281 | 0.374667 |
| DEPFSG | ILUFOV | 11 | 999 | 0.883503 | 0.588 | 0.670722 |
| DEPFSG | ILUFSG | 12 | 999 | 0.887089 | 0.614 | 0.682222 |
| DEPFSG | MYPFMG | 12 | 999 | 0.982925 | 0.396 | 0.482927 |
| DEPFSG | MYPFOV | 10 | 999 | 0.880046 | 0.631 | 0.695956 |
| DEPFSG | MYPFSG | 12 | 999 | 0.907659 | 0.603 | 0.677528 |
| DEPFSG | MYUFMG | 11 | 999 | 1.200251 | 0.184 | 0.274627 |
| DEPFSG | MYUFOV | 10 | 999 | 1.024725 | 0.408 | 0.491566 |
| DEPFSG | MYUFSG | 11 | 999 | 0.878047 | 0.593 | 0.673308 |
| DEPFSG | Pos. control | 11 | 999 | 5.453626 | 0.002 | 0.066667 |
| DEUFMG | DEUFOV | 12 | 999 | 2.495274 | 0.009 | 0.069231 |
| DEUFMG | DEUFSG | 12 | 999 | 0.806532 | 0.903 | 0.912121 |
| DEUFMG | ILPFMG | 11 | 999 | 1.464024 | 0.02 | 0.080769 |
| DEUFMG | ILPFOVG | 11 | 999 | 1.143464 | 0.189 | 0.27931 |
| DEUFMG | ILPFSG | 11 | 999 | 1.064867 | 0.362 | 0.450622 |
| DEUFMG | ILUFMG | 11 | 999 | 1.536182 | 0.007 | 0.066667 |
| DEUFMG | ILUFOV | 10 | 999 | 1.126038 | 0.182 | 0.274372 |
| DEUFMG | ILUFSG | 11 | 999 | 1.262243 | 0.063 | 0.136957 |
| DEUFMG | MYPFMG | 11 | 999 | 1.075502 | 0.297 | 0.392511 |
| DEUFMG | MYPFOV | 9 | 999 | 1.469869 | 0.013 | 0.072414 |
| DEUFMG | MYPFSG | 11 | 999 | 1.256227 | 0.07 | 0.143836 |
| DEUFMG | MYUFMG | 10 | 999 | 1.303373 | 0.058 | 0.130827 |
| DEUFMG | MYUFOV | 9 | 999 | 0.982216 | 0.521 | 0.605814 |
| DEUFMG | MYUFSG | 10 | 999 | 1.294072 | 0.05 | 0.120968 |
| DEUFMG | Pos. control | 10 | 999 | 6.845653 | 0.004 | 0.066667 |
| DEUFOV | DEUFSG | 12 | 999 | 2.22276 | 0.01 | 0.071429 |
| DEUFOV | ILPFMG | 11 | 999 | 1.997543 | 0.021 | 0.080769 |
| DEUFOV | ILPFOVG | 11 | 999 | 1.889642 | 0.027 | 0.093103 |
| DEUFOV | ILPFSG | 11 | 999 | 2.155587 | 0.012 | 0.072414 |
| DEUFOV | ILUFMG | 11 | 999 | 1.552806 | 0.032 | 0.098969 |
| DEUFOV | ILUFOV | 10 | 999 | 1.91556 | 0.029 | 0.093548 |
| DEUFOV | ILUFSG | 11 | 999 | 2.29774 | 0.007 | 0.066667 |
| DEUFOV | MYPFMG | 11 | 999 | 2.412545 | 0.01 | 0.071429 |
| DEUFOV | MYPFOV | 9 | 999 | 2.050025 | 0.017 | 0.076119 |
| DEUFOV | MYPFSG | 11 | 999 | 2.646723 | 0.005 | 0.066667 |
| DEUFOV | MYUFMG | 10 | 999 | 2.14285 | 0.02 | 0.080769 |
| DEUFOV | MYUFOV | 9 | 999 | 1.683326 | 0.039 | 0.104464 |
| DEUFOV | MYUFSG | 10 | 999 | 2.369737 | 0.005 | 0.066667 |
| DEUFOV | Pos. control | 10 | 999 | 2.76835 | 0.004 | 0.066667 |
| DEUFSG | ILPFMG | 11 | 999 | 1.2685 | 0.084 | 0.161538 |
| DEUFSG | ILPFOVG | 11 | 999 | 1.215481 | 0.168 | 0.257143 |
| DEUFSG | ILPFSG | 11 | 999 | 0.973819 | 0.517 | 0.603502 |
| DEUFSG | ILUFMG | 11 | 999 | 1.143271 | 0.15 | 0.240642 |
| DEUFSG | ILUFOV | 10 | 999 | 0.898972 | 0.646 | 0.702174 |
| DEUFSG | ILUFSG | 11 | 999 | 1.020385 | 0.358 | 0.4475 |
| DEUFSG | MYPFMG | 11 | 999 | 0.9584 | 0.544 | 0.625287 |
| DEUFSG | MYPFOV | 9 | 999 | 1.066738 | 0.343 | 0.434177 |
| DEUFSG | MYPFSG | 11 | 999 | 0.831381 | 0.886 | 0.897973 |
| DEUFSG | MYUFMG | 10 | 999 | 1.005454 | 0.421 | 0.5052 |
| DEUFSG | MYUFOV | 9 | 999 | 0.9882 | 0.499 | 0.58937 |
| DEUFSG | MYUFSG | 10 | 999 | 1.014953 | 0.405 | 0.489919 |
| DEUFSG | Pos. control | 10 | 999 | 5.753662 | 0.006 | 0.066667 |
| ILPFMG | ILPFOVG | 10 | 999 | 0.965404 | 0.555 | 0.635496 |
| ILPFMG | ILPFSG | 10 | 999 | 1.183648 | 0.114 | 0.2 |
| ILPFMG | ILUFMG | 10 | 999 | 1.164682 | 0.15 | 0.240642 |
| ILPFMG | ILUFOV | 9 | 999 | 1.10954 | 0.244 | 0.338889 |
| ILPFMG | ILUFSG | 10 | 999 | 1.119621 | 0.216 | 0.30566 |
| ILPFMG | MYPFMG | 10 | 999 | 1.21394 | 0.129 | 0.217877 |
| ILPFMG | MYPFOV | 8 | 999 | 1.474397 | 0.038 | 0.104464 |
| ILPFMG | MYPFSG | 10 | 999 | 1.492712 | 0.016 | 0.073846 |
| ILPFMG | MYUFMG | 9 | 999 | 1.123589 | 0.151 | 0.240957 |
| ILPFMG | MYUFOV | 8 | 999 | 0.808001 | 0.929 | 0.932107 |
| ILPFMG | MYUFSG | 9 | 999 | 1.492229 | 0.027 | 0.093103 |
| ILPFMG | Pos. control | 9 | 999 | 5.83194 | 0.004 | 0.066667 |
| ILPFOVG | ILPFSG | 10 | 999 | 1.222382 | 0.172 | 0.261929 |
| ILPFOVG | ILUFMG | 10 | 999 | 1.017511 | 0.466 | 0.552569 |
| ILPFOVG | ILUFOV | 9 | 999 | 1.048642 | 0.299 | 0.393421 |
| ILPFOVG | ILUFSG | 10 | 999 | 1.079378 | 0.261 | 0.359174 |
| ILPFOVG | MYPFMG | 10 | 999 | 1.246431 | 0.067 | 0.140559 |
| ILPFOVG | MYPFOV | 8 | 999 | 1.470878 | 0.016 | 0.073846 |
| ILPFOVG | MYPFSG | 10 | 999 | 1.430658 | 0.021 | 0.080769 |
| ILPFOVG | MYUFMG | 9 | 999 | 1.243353 | 0.173 | 0.262121 |
| ILPFOVG | MYUFOV | 8 | 999 | 0.997383 | 0.531 | 0.615058 |
| ILPFOVG | MYUFSG | 9 | 999 | 1.233131 | 0.107 | 0.192216 |
| ILPFOVG | Pos. control | 9 | 999 | 5.591793 | 0.008 | 0.066667 |
| ILPFSG | ILUFMG | 10 | 999 | 1.452108 | 0.028 | 0.093333 |
| ILPFSG | ILUFOV | 9 | 999 | 1.238967 | 0.114 | 0.2 |
| ILPFSG | ILUFSG | 10 | 999 | 1.379674 | 0.019 | 0.080769 |
| ILPFSG | MYPFMG | 10 | 999 | 1.043643 | 0.39 | 0.479508 |
| ILPFSG | MYPFOV | 8 | 999 | 1.584955 | 0.012 | 0.072414 |
| ILPFSG | MYPFSG | 10 | 999 | 1.380752 | 0.069 | 0.142759 |
| ILPFSG | MYUFMG | 9 | 999 | 1.546464 | 0.005 | 0.066667 |
| ILPFSG | MYUFOV | 8 | 999 | 1.102139 | 0.228 | 0.31814 |
| ILPFSG | MYUFSG | 9 | 999 | 1.459825 | 0.038 | 0.104464 |
| ILPFSG | Pos. control | 9 | 999 | 6.49047 | 0.013 | 0.072414 |
| ILUFMG | ILUFOV | 9 | 999 | 1.005396 | 0.393 | 0.481224 |
| ILUFMG | ILUFSG | 10 | 999 | 1.11619 | 0.224 | 0.314019 |
| ILUFMG | MYPFMG | 10 | 999 | 1.25198 | 0.081 | 0.158824 |
| ILUFMG | MYPFOV | 8 | 999 | 1.11087 | 0.195 | 0.285366 |
| ILUFMG | MYPFSG | 10 | 999 | 1.145174 | 0.128 | 0.217877 |
| ILUFMG | MYUFMG | 9 | 999 | 1.164162 | 0.135 | 0.222527 |
| ILUFMG | MYUFOV | 8 | 999 | 0.920024 | 0.637 | 0.696 |
| ILUFMG | MYUFSG | 9 | 999 | 1.123348 | 0.201 | 0.291304 |
| ILUFMG | Pos. control | 9 | 999 | 3.969174 | 0.008 | 0.066667 |
| ILUFOV | ILUFSG | 9 | 999 | 0.865204 | 0.851 | 0.868367 |
| ILUFOV | MYPFMG | 9 | 999 | 1.159153 | 0.156 | 0.245026 |
| ILUFOV | MYPFOV | 7 | 999 | 0.867643 | 0.875 | 0.889831 |
| ILUFOV | MYPFSG | 9 | 999 | 0.978876 | 0.506 | 0.592969 |
| ILUFOV | MYUFMG | 8 | 999 | 1.135585 | 0.12 | 0.206897 |
| ILUFOV | MYUFOV | 7 | 999 | 0.945165 | 0.692 | 0.735211 |
| ILUFOV | MYUFSG | 8 | 999 | 0.824095 | 0.823 | 0.848454 |
| ILUFOV | Pos. control | 8 | 999 | 5.268283 | 0.028 | 0.093333 |
| ILUFSG | MYPFMG | 10 | 999 | 1.042199 | 0.291 | 0.386283 |
| ILUFSG | MYPFOV | 8 | 999 | 1.030024 | 0.354 | 0.444351 |
| ILUFSG | MYPFSG | 10 | 999 | 1.012412 | 0.342 | 0.434177 |
| ILUFSG | MYUFMG | 9 | 999 | 1.244979 | 0.059 | 0.13209 |
| ILUFSG | MYUFOV | 8 | 999 | 0.935136 | 0.683 | 0.731786 |
| ILUFSG | MYUFSG | 9 | 999 | 1.068613 | 0.31 | 0.402597 |
| ILUFSG | Pos. control | 9 | 999 | 5.850154 | 0.007 | 0.066667 |
| MYPFMG | MYPFOV | 8 | 999 | 1.061146 | 0.277 | 0.370982 |
| MYPFMG | MYPFSG | 10 | 999 | 1.161018 | 0.134 | 0.222099 |
| MYPFMG | MYUFMG | 9 | 999 | 1.427145 | 0.039 | 0.104464 |
| MYPFMG | MYUFOV | 8 | 999 | 0.915343 | 0.677 | 0.727957 |
| MYPFMG | MYUFSG | 9 | 999 | 1.166075 | 0.163 | 0.253368 |
| MYPFMG | Pos. control | 9 | 999 | 6.496129 | 0.004 | 0.066667 |
| MYPFOV | MYPFSG | 8 | 999 | 0.942966 | 0.611 | 0.681413 |
| MYPFOV | MYUFMG | 7 | 999 | 1.443288 | 0.029 | 0.093548 |
| MYPFOV | MYUFOV | 6 | 999 | 1.382395 | 0.114 | 0.2 |
| MYPFOV | MYUFSG | 7 | 999 | 1.081791 | 0.303 | 0.396943 |
| MYPFOV | Pos. control | 7 | 999 | 5.263533 | 0.03 | 0.094737 |
| MYPFSG | MYUFMG | 9 | 999 | 1.280806 | 0.065 | 0.139286 |
| MYPFSG | MYUFOV | 8 | 999 | 1.054425 | 0.34 | 0.434043 |
| MYPFSG | MYUFSG | 9 | 999 | 0.841561 | 0.816 | 0.844138 |
| MYPFSG | Pos. control | 9 | 999 | 6.304895 | 0.006 | 0.066667 |
| MYUFMG | MYUFOV | 7 | 999 | 1.176985 | 0.146 | 0.238043 |
| MYUFMG | MYUFSG | 8 | 999 | 1.367505 | 0.055 | 0.126923 |
| MYUFMG | Pos. control | 8 | 999 | 5.904442 | 0.027 | 0.093103 |
| MYUFOV | MYUFSG | 7 | 999 | 1.092374 | 0.325 | 0.418455 |
| MYUFOV | Pos. control | 7 | 999 | 5.164927 | 0.035 | 0.100962 |
| MYUFSG | Pos. control | 8 | 999 | 6.183192 | 0.038 | 0.104464 |

Additional File 13: Unweighted UniFrac diversity distances of *Amblyomma americanum* field-collected ticks (pairwise).

| Group 1 | Group 2 | Sample size | Permutations | pseudo-F | p-value | q-value |
| --- | --- | --- | --- | --- | --- | --- |
| DEPFMG | DEPFOV | 14 | 999 | 0.584023 | 0.894 | 0.906061 |
| DEPFMG | DEPFSG | 14 | 999 | 0.852208 | 0.498 | 0.574615 |
| DEPFOV | DEPFSG | 14 | 999 | 0.96291 | 0.439 | 0.520553 |
| DEPFMG | DEUFMG | 13 | 999 | 4.91675 | 0.009 | 0.058696 |
| DEPFOV | DEUFMG | 13 | 999 | 5.782557 | 0.002 | 0.051429 |
| DEPFSG | DEUFMG | 13 | 999 | 3.371387 | 0.014 | 0.068182 |
| DEPFMG | DEUFOV | 13 | 999 | 12.53895 | 0.001 | 0.051429 |
| DEPFOV | DEUFOV | 13 | 999 | 14.68384 | 0.002 | 0.051429 |
| DEPFSG | DEUFOV | 13 | 999 | 11.53606 | 0.002 | 0.051429 |
| DEUFMG | DEUFOV | 12 | 999 | 8.121403 | 0.003 | 0.051429 |
| DEPFMG | DEUFSG | 13 | 999 | 2.19882 | 0.082 | 0.15092 |
| DEPFOV | DEUFSG | 13 | 999 | 2.210631 | 0.036 | 0.096522 |
| DEPFSG | DEUFSG | 13 | 999 | 1.15412 | 0.288 | 0.378879 |
| DEUFMG | DEUFSG | 12 | 999 | 1.248643 | 0.229 | 0.318056 |
| DEUFOV | DEUFSG | 12 | 999 | 8.298898 | 0.004 | 0.051429 |
| DEPFMG | ILPFMG | 12 | 999 | 1.584518 | 0.154 | 0.240625 |
| DEPFOV | ILPFMG | 12 | 999 | 1.938321 | 0.039 | 0.099153 |
| DEPFSG | ILPFMG | 12 | 999 | 1.546152 | 0.14 | 0.222222 |
| DEUFMG | ILPFMG | 11 | 999 | 6.456048 | 0.004 | 0.051429 |
| DEUFOV | ILPFMG | 11 | 999 | 13.5544 | 0.004 | 0.051429 |
| DEUFSG | ILPFMG | 11 | 999 | 2.098937 | 0.062 | 0.125676 |
| DEPFMG | ILPFOVG | 12 | 999 | 1.688826 | 0.147 | 0.232105 |
| DEPFOV | ILPFOVG | 12 | 999 | 1.899834 | 0.056 | 0.119149 |
| DEPFSG | ILPFOVG | 12 | 999 | 1.157959 | 0.304 | 0.391416 |
| DEUFMG | ILPFOVG | 11 | 999 | 1.859195 | 0.089 | 0.158929 |
| DEUFOV | ILPFOVG | 11 | 999 | 6.094186 | 0.018 | 0.076056 |
| DEUFSG | ILPFOVG | 11 | 999 | 0.613849 | 0.757 | 0.791289 |
| ILPFMG | ILPFOVG | 10 | 999 | 1.075714 | 0.355 | 0.445607 |
| DEPFMG | ILPFSG | 12 | 999 | 1.390269 | 0.201 | 0.289904 |
| DEPFOV | ILPFSG | 12 | 999 | 1.762777 | 0.071 | 0.136538 |
| DEPFSG | ILPFSG | 12 | 999 | 0.994005 | 0.384 | 0.473361 |
| DEUFMG | ILPFSG | 11 | 999 | 3.592164 | 0.013 | 0.068182 |
| DEUFOV | ILPFSG | 11 | 999 | 11.23286 | 0.005 | 0.051429 |
| DEUFSG | ILPFSG | 11 | 999 | 0.858775 | 0.489 | 0.566409 |
| ILPFMG | ILPFSG | 10 | 999 | 0.874195 | 0.553 | 0.623684 |
| ILPFOVG | ILPFSG | 10 | 999 | 0.660019 | 0.717 | 0.760071 |
| DEPFMG | ILUFMG | 12 | 999 | 1.85565 | 0.084 | 0.152727 |
| DEPFOV | ILUFMG | 12 | 999 | 2.221697 | 0.029 | 0.089109 |
| DEPFSG | ILUFMG | 12 | 999 | 1.454555 | 0.173 | 0.263452 |
| DEUFMG | ILUFMG | 11 | 999 | 2.922314 | 0.015 | 0.068182 |
| DEUFOV | ILUFMG | 11 | 999 | 5.852673 | 0.011 | 0.067347 |
| DEUFSG | ILUFMG | 11 | 999 | 1.380409 | 0.2 | 0.289855 |
| ILPFMG | ILUFMG | 10 | 999 | 1.008324 | 0.427 | 0.510359 |
| ILPFOVG | ILUFMG | 10 | 999 | 0.705885 | 0.57 | 0.633333 |
| ILPFSG | ILUFMG | 10 | 999 | 1.260503 | 0.254 | 0.346364 |
| DEPFMG | ILUFOV | 11 | 999 | 1.53584 | 0.163 | 0.252062 |
| DEPFOV | ILUFOV | 11 | 999 | 1.87718 | 0.057 | 0.11958 |
| DEPFSG | ILUFOV | 11 | 999 | 1.193139 | 0.237 | 0.326147 |
| DEUFMG | ILUFOV | 10 | 999 | 3.488347 | 0.008 | 0.054545 |
| DEUFOV | ILUFOV | 10 | 999 | 7.874216 | 0.012 | 0.067925 |
| DEUFSG | ILUFOV | 10 | 999 | 1.529539 | 0.112 | 0.187709 |
| ILPFMG | ILUFOV | 9 | 999 | 1.319946 | 0.134 | 0.216129 |
| ILPFOVG | ILUFOV | 9 | 999 | 0.903632 | 0.582 | 0.642857 |
| ILPFSG | ILUFOV | 9 | 999 | 1.329751 | 0.192 | 0.280976 |
| ILUFMG | ILUFOV | 9 | 999 | 0.795375 | 0.698 | 0.747857 |
| DEPFMG | ILUFSG | 12 | 999 | 0.765795 | 0.583 | 0.642857 |
| DEPFOV | ILUFSG | 12 | 999 | 0.69779 | 0.799 | 0.826552 |
| DEPFSG | ILUFSG | 12 | 999 | 1.065634 | 0.283 | 0.37533 |
| DEUFMG | ILUFSG | 11 | 999 | 4.969028 | 0.002 | 0.051429 |
| DEUFOV | ILUFSG | 11 | 999 | 13.10206 | 0.005 | 0.051429 |
| DEUFSG | ILUFSG | 11 | 999 | 1.760429 | 0.096 | 0.170175 |
| ILPFMG | ILUFSG | 10 | 999 | 1.530008 | 0.101 | 0.172159 |
| ILPFOVG | ILUFSG | 10 | 999 | 1.122228 | 0.284 | 0.37533 |
| ILPFSG | ILUFSG | 10 | 999 | 1.48202 | 0.109 | 0.184746 |
| ILUFMG | ILUFSG | 10 | 999 | 1.37864 | 0.216 | 0.304225 |
| ILUFOV | ILUFSG | 9 | 999 | 1.557378 | 0.084 | 0.152727 |
| DEPFMG | MYPFMG | 12 | 999 | 0.94216 | 0.385 | 0.473361 |
| DEPFOV | MYPFMG | 12 | 999 | 1.020977 | 0.429 | 0.510714 |
| DEPFSG | MYPFMG | 12 | 999 | 0.893828 | 0.513 | 0.589655 |
| DEUFMG | MYPFMG | 11 | 999 | 5.021665 | 0.005 | 0.051429 |
| DEUFOV | MYPFMG | 11 | 999 | 14.07625 | 0.004 | 0.051429 |
| DEUFSG | MYPFMG | 11 | 999 | 1.638565 | 0.11 | 0.185393 |
| ILPFMG | MYPFMG | 10 | 999 | 1.285021 | 0.178 | 0.265672 |
| ILPFOVG | MYPFMG | 10 | 999 | 1.255064 | 0.174 | 0.263636 |
| ILPFSG | MYPFMG | 10 | 999 | 0.894515 | 0.567 | 0.632342 |
| ILUFMG | MYPFMG | 10 | 999 | 1.608558 | 0.136 | 0.218182 |
| ILUFOV | MYPFMG | 9 | 999 | 1.424022 | 0.1 | 0.172159 |
| ILUFSG | MYPFMG | 10 | 999 | 1.155985 | 0.293 | 0.378879 |
| DEPFMG | MYPFOV | 10 | 999 | 0.597109 | 0.903 | 0.90906 |
| DEPFOV | MYPFOV | 10 | 999 | 0.748481 | 0.745 | 0.781469 |
| DEPFSG | MYPFOV | 10 | 999 | 1.347438 | 0.223 | 0.312617 |
| DEUFMG | MYPFOV | 9 | 999 | 6.847569 | 0.012 | 0.067925 |
| DEUFOV | MYPFOV | 9 | 999 | 12.81997 | 0.012 | 0.067925 |
| DEUFSG | MYPFOV | 9 | 999 | 2.827252 | 0.022 | 0.076744 |
| ILPFMG | MYPFOV | 8 | 999 | 1.911117 | 0.039 | 0.099153 |
| ILPFOVG | MYPFOV | 8 | 999 | 1.948508 | 0.043 | 0.1032 |
| ILPFSG | MYPFOV | 8 | 999 | 2.147552 | 0.03 | 0.089109 |
| ILUFMG | MYPFOV | 8 | 999 | 1.69661 | 0.089 | 0.158929 |
| ILUFOV | MYPFOV | 7 | 999 | 1.791153 | 0.058 | 0.12 |
| ILUFSG | MYPFOV | 8 | 999 | 1.06498 | 0.322 | 0.411064 |
| MYPFMG | MYPFOV | 8 | 999 | 1.440722 | 0.18 | 0.267327 |
| DEPFMG | MYPFSG | 12 | 999 | 0.698737 | 0.705 | 0.75 |
| DEPFOV | MYPFSG | 12 | 999 | 0.966481 | 0.472 | 0.550973 |
| DEPFSG | MYPFSG | 12 | 999 | 1.797349 | 0.077 | 0.144375 |
| DEUFMG | MYPFSG | 11 | 999 | 9.219947 | 0.002 | 0.051429 |
| DEUFOV | MYPFSG | 11 | 999 | 19.48337 | 0.006 | 0.051429 |
| DEUFSG | MYPFSG | 11 | 999 | 3.42937 | 0.006 | 0.051429 |
| ILPFMG | MYPFSG | 10 | 999 | 2.278377 | 0.021 | 0.076744 |
| ILPFOVG | MYPFSG | 10 | 999 | 2.460986 | 0.015 | 0.068182 |
| ILPFSG | MYPFSG | 10 | 999 | 2.604627 | 0.03 | 0.089109 |
| ILUFMG | MYPFSG | 10 | 999 | 2.340295 | 0.04 | 0.099174 |
| ILUFOV | MYPFSG | 9 | 999 | 2.681286 | 0.017 | 0.073913 |
| ILUFSG | MYPFSG | 10 | 999 | 1.143607 | 0.292 | 0.378879 |
| MYPFMG | MYPFSG | 10 | 999 | 1.754854 | 0.058 | 0.12 |
| MYPFOV | MYPFSG | 8 | 999 | 0.791392 | 0.861 | 0.88157 |
| DEPFMG | MYUFMG | 11 | 999 | 2.023291 | 0.097 | 0.170175 |
| DEPFOV | MYUFMG | 11 | 999 | 2.256941 | 0.036 | 0.096522 |
| DEPFSG | MYUFMG | 11 | 999 | 1.25347 | 0.252 | 0.345205 |
| DEUFMG | MYUFMG | 10 | 999 | 1.958938 | 0.047 | 0.106015 |
| DEUFOV | MYUFMG | 10 | 999 | 8.672716 | 0.015 | 0.068182 |
| DEUFSG | MYUFMG | 10 | 999 | 0.742613 | 0.633 | 0.688043 |
| ILPFMG | MYUFMG | 9 | 999 | 1.986002 | 0.101 | 0.172159 |
| ILPFOVG | MYUFMG | 9 | 999 | 0.781875 | 0.544 | 0.616981 |
| ILPFSG | MYUFMG | 9 | 999 | 1.299648 | 0.216 | 0.304225 |
| ILUFMG | MYUFMG | 9 | 999 | 1.304113 | 0.21 | 0.3 |
| ILUFOV | MYUFMG | 8 | 999 | 1.307834 | 0.228 | 0.318056 |
| ILUFSG | MYUFMG | 9 | 999 | 2.107248 | 0.044 | 0.103937 |
| MYPFMG | MYUFMG | 9 | 999 | 1.905192 | 0.086 | 0.155422 |
| MYPFOV | MYUFMG | 7 | 999 | 2.889485 | 0.046 | 0.104545 |
| MYPFSG | MYUFMG | 9 | 999 | 3.508994 | 0.022 | 0.076744 |
| DEPFMG | MYUFOV | 10 | 999 | 0.741207 | 0.545 | 0.616981 |
| DEPFOV | MYUFOV | 10 | 999 | 1.010559 | 0.419 | 0.5028 |
| DEPFSG | MYUFOV | 10 | 999 | 1.013484 | 0.404 | 0.490688 |
| DEUFMG | MYUFOV | 9 | 999 | 2.990495 | 0.026 | 0.084783 |
| DEUFOV | MYUFOV | 9 | 999 | 6.822986 | 0.015 | 0.068182 |
| DEUFSG | MYUFOV | 9 | 999 | 1.216123 | 0.282 | 0.37533 |
| ILPFMG | MYUFOV | 8 | 999 | 1.022761 | 0.382 | 0.473361 |
| ILPFOVG | MYUFOV | 8 | 999 | 0.716275 | 0.704 | 0.75 |
| ILPFSG | MYUFOV | 8 | 999 | 0.945762 | 0.414 | 0.500806 |
| ILUFMG | MYUFOV | 8 | 999 | 0.895711 | 0.461 | 0.540234 |
| ILUFOV | MYUFOV | 7 | 999 | 0.818667 | 0.65 | 0.701439 |
| ILUFSG | MYUFOV | 8 | 999 | 1.050929 | 0.352 | 0.443697 |
| MYPFMG | MYUFOV | 8 | 999 | 0.836434 | 0.585 | 0.642857 |
| MYPFOV | MYUFOV | 6 | 999 | 1.238493 | 0.399 | 0.486585 |
| MYPFSG | MYUFOV | 8 | 999 | 1.313027 | 0.213 | 0.302844 |
| MYUFMG | MYUFOV | 7 | 999 | 1.128577 | 0.328 | 0.416949 |
| DEPFMG | MYUFSG | 11 | 999 | 0.618084 | 0.838 | 0.860959 |
| DEPFOV | MYUFSG | 11 | 999 | 0.512668 | 0.938 | 0.941137 |
| DEPFSG | MYUFSG | 11 | 999 | 0.909061 | 0.46 | 0.540234 |
| DEUFMG | MYUFSG | 10 | 999 | 5.677972 | 0.005 | 0.051429 |
| DEUFOV | MYUFSG | 10 | 999 | 12.59033 | 0.006 | 0.051429 |
| DEUFSG | MYUFSG | 10 | 999 | 1.964786 | 0.061 | 0.125342 |
| ILPFMG | MYUFSG | 9 | 999 | 1.381484 | 0.073 | 0.137736 |
| ILPFOVG | MYUFSG | 9 | 999 | 1.247424 | 0.181 | 0.267488 |
| ILPFSG | MYUFSG | 9 | 999 | 1.3816 | 0.148 | 0.232461 |
| ILUFMG | MYUFSG | 9 | 999 | 1.162 | 0.331 | 0.418987 |
| ILUFOV | MYUFSG | 8 | 999 | 1.449195 | 0.121 | 0.198361 |
| ILUFSG | MYUFSG | 9 | 999 | 0.818172 | 0.526 | 0.6 |
| MYPFMG | MYUFSG | 9 | 999 | 1.061229 | 0.396 | 0.484898 |
| MYPFOV | MYUFSG | 7 | 999 | 0.8912 | 0.525 | 0.6 |
| MYPFSG | MYUFSG | 9 | 999 | 0.987966 | 0.441 | 0.520866 |
| MYUFMG | MYUFSG | 8 | 999 | 2.356613 | 0.049 | 0.108889 |
| MYUFOV | MYUFSG | 7 | 999 | 0.861245 | 0.56 | 0.629104 |
| DEPFMG | Pos. control | 11 | 999 | 19.8367 | 0.003 | 0.051429 |
| DEPFOV | Pos. control | 11 | 999 | 25.25117 | 0.004 | 0.051429 |
| DEPFSG | Pos. control | 11 | 999 | 22.56159 | 0.006 | 0.051429 |
| DEUFMG | Pos. control | 10 | 999 | 35.04203 | 0.006 | 0.051429 |
| DEUFOV | Pos. control | 10 | 999 | 9.363714 | 0.01 | 0.06383 |
| DEUFSG | Pos. control | 10 | 999 | 22.19985 | 0.004 | 0.051429 |
| ILPFMG | Pos. control | 9 | 999 | 39.88974 | 0.008 | 0.054545 |
| ILPFOVG | Pos. control | 9 | 999 | 15.93339 | 0.022 | 0.076744 |
| ILPFSG | Pos. control | 9 | 999 | 34.87535 | 0.006 | 0.051429 |
| ILUFMG | Pos. control | 9 | 999 | 13.11005 | 0.018 | 0.076056 |
| ILUFOV | Pos. control | 8 | 999 | 24.63092 | 0.021 | 0.076744 |
| ILUFSG | Pos. control | 9 | 999 | 29.97207 | 0.004 | 0.051429 |
| MYPFMG | Pos. control | 9 | 999 | 36.01715 | 0.013 | 0.068182 |
| MYPFOV | Pos. control | 7 | 999 | 33.79385 | 0.03 | 0.089109 |
| MYPFSG | Pos. control | 9 | 999 | 45.61494 | 0.008 | 0.054545 |
| MYUFMG | Pos. control | 8 | 999 | 33.33147 | 0.027 | 0.087097 |
| MYUFOV | Pos. control | 7 | 999 | 19.8991 | 0.026 | 0.084783 |
| MYUFSG | Pos. control | 8 | 999 | 34.67915 | 0.029 | 0.089109 |
